# Sex differences in the transcriptome of extracellular vesicles secreted by fetal neural stem cells and effects of chronic alcohol exposure

**DOI:** 10.1186/s13293-023-00503-0

**Published:** 2023-04-15

**Authors:** Dae D. Chung, Amanda H. Mahnke, Marisa R. Pinson, Nihal A. Salem, Michael S. Lai, Natalie P. Collins, Andrew E. Hillhouse, Rajesh C. Miranda

**Affiliations:** 1 School of Medicine, Department of Neuroscience and Experimental Therapeutics, Medical Research and Education, Texas A&M University Health Science Center, Building 8447 Riverside Parkway, Bryan, TX 77807-3260 USA; 2 Women’s Health in Neuroscience, Texas A&M University Health Science Center, Bryan, TX USA; 3 Texas A&M Institute for Genome Sciences and Society, Texas A&M University, College Station, TX 77843 USA

**Keywords:** Prenatal alcohol exposure, Fetal sex, Ethanol, FASD, WGCNA, Consensus WGCNA

## Abstract

**Background:**

Prenatal alcohol (ethanol) exposure (PAE) results in brain growth restriction, in part, by reprogramming self-renewal and maturation of fetal neural stem cells (NSCs) during neurogenesis. We recently showed that ethanol resulted in enrichment of both proteins and pro-maturation microRNAs in sub-200-nm-sized extracellular vesicles (EVs) secreted by fetal NSCs. Moreover, EVs secreted by ethanol-exposed NSCs exhibited diminished efficacy in controlling NSC metabolism and maturation. Here we tested the hypothesis that ethanol may also influence the packaging of RNAs into EVs from cell-of-origin NSCs.

**Methods:**

Sex-specified fetal murine iso-cortical neuroepithelia from three separate pregnancies were maintained ex vivo, as neurosphere cultures to model the early neurogenic niche. EVs were isolated by ultracentrifugation from NSCs exposed to a dose range of ethanol. RNA from paired EV and cell-of-origin NSC samples was processed for ribosomal RNA-depleted RNA sequencing. Differential expression analysis and exploratory weighted gene co-expression network analysis (WGCNA) identified candidate genes and gene networks that were drivers of alterations to the transcriptome of EVs relative to cells.

**Results:**

The RNA content of EVs differed significantly from cell-of-origin NSCs. Biological sex contributed to unique transcriptome variance in EV samples, where > 75% of the most variant transcripts were also sex-variant in EVs but not in cell-of-origin NSCs. WGCNA analysis also identified sex-dependent enrichment of pathways, including dopamine receptor binding and ectoderm formation in female EVs and cell-substrate adhesion in male EVs, with the top significant DEGs from differential analysis of overall individual gene expressions, i.e., *Arhgap15,* enriched in female EVs, and *Cenpa,* enriched in male EVs*,* also serving as WCGNA hub genes of sex-biased EV WGCNA clusters. In addition to the baseline RNA content differences, ethanol exposure resulted in a significant dose-dependent change in transcript expression in both EVs and cell-of-origin NSCs that predominantly altered sex-invariant RNAs. Moreover, at the highest dose, ~ 73% of significantly altered RNAs were enriched in EVs, but depleted in NSCs.

**Conclusions:**

The EV transcriptome is distinctly different from, and more sex-variant than, the transcriptome of cell-of-origin NSCs. Ethanol, a common teratogen, results in dose-dependent sorting of RNA transcripts from NSCs to EVs which may reprogram the EV-mediated endocrine environment during neurogenesis.

**Supplementary Information:**

The online version contains supplementary material available at 10.1186/s13293-023-00503-0.

## Highlights

- The RNA content of EVs differ significantly from their cell-of-origin NSCs.
- Biological sex contributes to significant differences in the transcriptome of EVs.
- Most sex differences observed in EVs were not present in parent-of-origin NSCs, suggesting sex-dependent sorting of RNA into EVs.
- Ethanol exposure resulted in specific RNA transcripts being enriched in secreted EVs, while being depleting in cell-of-origin NSCs, suggesting that cell stress may alter the sorting of RNA from cells to EVs.
- Transcripts enriched in EVs, following ethanol exposure of NSCs, encode proteins selectively overrepresented in biological pathways like mRNA splicing and transport.

**Supplementary Information:**

The online version contains supplementary material available at 10.1186/s13293-023-00503-0.

## Background

Prenatal alcohol exposure (PAE) is common. A meta-analysis study estimated that the prevalence of PAE was 9.8% globally, and 11.2% in the Americas [1]. Alcohol readily crosses the placenta to reach approximate blood alcohol levels equal to that in maternal circulation, and is therefore in a position to directly alter fetal brain development [2]. Its outcome, ‘Fetal Alcohol Spectrum Disorders’ (FASD), a spectrum of brain-based disabilities, craniofacial anomalies, and growth deficits [3], is estimated to affect 1.13 to 9.85% of school-aged children in the US [4]. Studies in animal models [5–7] and human populations [8–11] show that developmental exposure to ethanol/alcohol can result in decreased brain growth and microcephaly. Our studies have focused on the vulnerability of fetal neural stem cells (NSCs), which, during the mid-first through second trimester of pregnancy, generate most of the neurons of adult brain regions like the cerebral cortex [12]. Previous studies, including our published data, document that ethanol exposure does not cause apoptosis of fetal cerebral cortical NSCs, but rather causes rapid proliferation and transit amplification of NSCs, which results in premature maturation and depletion of NSCs, thereby reducing the pool of available NSCs for subsequent neurogenic cycles [13–22]. However, the mechanisms which mediate the effects of PAE on NSC growth are poorly understood. Recent studies have focused on the mediating role of a class of nanometer-sized extracellular vesicles (EVs), which represent a novel means of intercellular communication, and paracrine and endocrine transfer of proteins and nucleic acids to other cells without a direct cell-to-cell contact [23–31].

NSCs are highly secretory cells that both secrete and endocytose EVs effectively [32–34]. Recently, we showed that incubating recipient NSCs and differentiating neural precursors with EVs purified from donor NSCs decreased rates of oxidative metabolism in both immature NSCs and maturing neuronal progenitors [32]. We also observed decreased numbers of recipient NSCs in S-phase and, in more mature progenitors, decreased expression of the oligodendrocyte marker Olig2 and increased glycolysis. Moreover, retroviral-like proteins like PEG10 and PNMA2 are packaged in EVs [33] and may confer apoptosis-resistance to NSCs. These data suggest that EVs secreted by NSCs can influence metabolism, survival, and maturation of recipient NSCs as well as more mature progeny cells.

We also previously showed that exposing NSCs to ethanol alters the function of EVs as well as their miRNA and protein content. Purified EVs from ethanol-treated NSCs exhibited diminished effect on metabolism and cell cycle of recipient NSCs when compared to EVs from untreated control NSCs. Ethanol exposure also resulted in enrichment of miRNAs, such as miR-140-3p, in EVs, which may mediate the pro-maturation effects of ethanol on NSCs [21]. Our recent data also showed that ethanol has a significant and EV-specific impact on the proteome of EVs secreted by NSCs. Ethanol exposure of cell-of-origin NSCs resulted in an enrichment of RNA-binding chaperone proteins in EVs concomitant with their depletion in cell-of-origin NSCs [32]. These data suggested a hypothesis that ethanol exposure also affects the transfer of RNAs, aside from miRNAs, from NSCs to EVs.

A few studies in human populations [35–38] have documented sex differences in FASD presentation and other studies in rodent models [13, 39] have described prenatal sex differences due to PAE. Moreover, a few studies have also documented sex differences in EVs secreted from neural and non-neural tissues [40–43]. Collectively the research suggests that biological sex may contribute to both the effects of PAE and to the content of EVs that are positioned to mediate some effects of PAE. Therefore, in this study, we assessed the transcriptome of EVs derived from male or female sex-specified fetal NSCs and compared the EV transcriptome to that of matched cell-of-origin NSCs. We analyzed our previously published single cell RNAseq (scRNAseq) data from ethanol-exposed GD 14.5 mouse cerebral cortex [13], to validate fetal NSCs as the source of ethanol-sensitive RNA transcripts secreted in EVs. Our analyses showed that the EV transcriptome and associated biological pathways diverge substantially from that of the cell-of-origin NSCs. Moreover, we found that the biological sex of the fetus contributed more to the variability of EV transcriptome than to the cell-of-origin transcriptome. This is in contrast to our previous EV proteomic analysis [32] from the same EVs which found only minimal sex differences. Here we also report that EVs exhibit sex differences that are not observed in cell-of-origin NSCs. Together, these data suggest the presence of a hitherto unsuspected, sex-dependent sorting mechanism that results in the divergence of the transcriptome, but not proteome, of EVs from cell-of-origin NSCs. As we previously observed with shifts in the EV proteome [32], exposing cell-of-origin NSCs to ethanol resulted in a dose-related enrichment of RNA transcripts in EVs at the expense of cell-of-origin NSCs. Moreover, transcripts enriched in EVs predominantly encoded nuclear proteins and were overrepresented in specific biological pathways, including the processing and splicing of pre-mRNAs, that may influence the biology of recipient cells. These data further indicate that environmental perturbations can also contribute to the sorting of RNA into EVs.

## Materials and methods

### Ex vivo fetal mouse neurosphere culture model

**Fig. 1:**
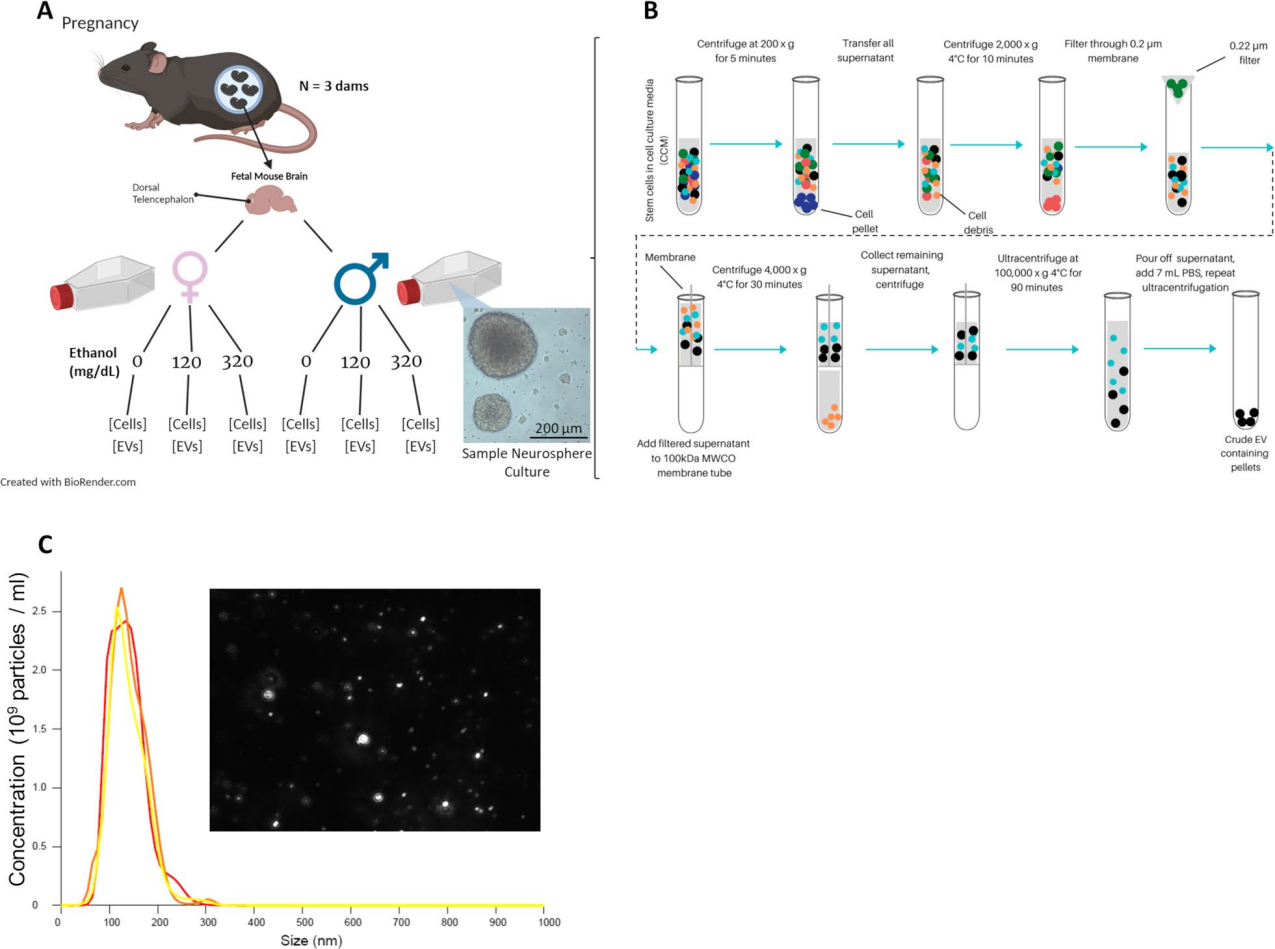
Schematic of the Study Protocol Ex Vivo Fetal Mouse Neurosphere Culture Model with Ethanol Treatment and EV Concentration and Characterization. **A** NSCs were collected from three separate pregnancies from fetal mouse dorsal telencephalon, segregated by genetic sex, and propagated ex vivo as neurosphere cultures (inset photomicrograph; scale bar, 200 μm). Cultures were treated with control medium or with culture medium containing ethanol (120 or 320 mg/dL), cells were collected and culture-conditioned medium was processed for EV separation and concentration. **B** Cell culture supernatant was processed by ultracentrifugation protocol for EV separation and concentration (see detailed methods). **C** Frequency Distribution of EV diameter. Inset depicts Nanosight image of EVs derived from NSCs

C57BL/6J (Ai14) mice (Jackson Laboratories; Catalog # 007914) were bred in-house, and time-mated overnight at the start of the dark phase of the light–dark cycle. The following morning was defined as gestational day (GD) 0.5. PAE in the 1st trimester can result in microcephaly in human populations [44–46] suggesting that ethanol interferes with development of the telencephalon and specifically with neurogenesis [15, 18, 20]. To model early neuronal development effects of ethanol, ex vivo neurosphere cultures were generated from NSCs collected from acutely dissociated GD 12.5 fetal mouse dorsal telencephalic neuroepithelium that corresponds to the future isocortex, from three separate pregnancies [18, 20]. Murine GD 12.5 represents the initial period of cortical plate neurogenesis, equivalent to the latter half of the 1st trimester of human fetal development [12, 47], where stem cells of the murine fetal ventricular zone (VZ) begin to generate the neurons and deep-to-superficial laminar organization of the cortical plate [48–50]. Since the stem cells of the VZ have the potential to generate the entire cortical plate at this period, we used cells derived from this time period, to broadly model cortical plate neurogenesis. As we have previously reported, fetal sex was determined at the time of collection [13]. Briefly, alkaline lysis was used on fetal tissue samples to obtain genomic DNA, followed by a rapid qPCR protocol with primers to detect repetitive sequences on X and Y chromosomes to determine the fetal sex [51, 52]. Male and female sex-specific cultures were generated by pooling dorsal telencephalic cortical neuroepithelial tissues from a single pregnancy by sex. Three separate biological replicates for each fetal sex were generated by collecting and dissociating sex-specified neuroepithelia from three separate pregnancies into single cell suspensions, and maintaining these as non-adherent neurospheres in serum-free mitogenic media, as previously published [18, 53, 54] (Fig. 1A). Neurosphere cultures between passages 7 to 10 were used in this study. All animal procedures were performed in accordance with the Texas A&M University Institutional Animal Care and Use Committee guidelines and approval.

### Ethanol treatment

Dispersed single-cell NSCs were seeded in T75 flasks at a density of 4 × 10^5^ cells/mL in 10 mL of culture media per flask. Cell viability (~ 75 to 85% viability) and number were measured using the Invitrogen Countess Automated Cell Counter (Invitrogen; Catalog # C10227; Carlsbad, CA/USA). Four flasks were defined as a single sample. Ethanol can be consumed and tolerated by humans at doses in excess of 100 mM, and also exhibits its psychological effects in the millimolar range [55, 56]. Therefore, we used a socio-culturally relevant dose-range of ethanol that is associated with risky drinking in humans. To model ethanol exposure levels from abstinence to the heavy exposure attainable by individuals with alcohol use disorders, each sample was randomly assigned to one of three ethanol treatment conditions: 0 mg/dL (control), 120 mg/dL (26 mM, moderate, level achievable by individuals that binge drink), or 320 mg/dL (70 mM, high, level achievable by individuals with chronic alcohol use) [55]. Ethanol (190 proof grain alcohol) was diluted into fresh culture medium and ethanol-naïve media was replaced with fresh experimental media. In addition, to model a period equivalent to in vivo exposure during the period of cortical plate neurogenesis in a mouse [50, 57], neurosphere cultures were exposed to control medium or medium with ethanol, for five days, with media replacement on day 3. To prevent ethanol loss in the culture medium throughout the experiment and mimic chronic alcohol exposure in fetus, all flasks were tightly capped with phenolic caps and sealed with parafilm. For each experiment, gas chromatography was used to verify ethanol concentrations in culture-conditioned medium. The measured ethanol dose range, from moderate (100–134 mg/dL; 22 Mm–29 mM) to high (250–380 mg/dL; 54 mM–82 mM), was consistent with our previously published studies [14, 18, 57, 58]. On day 5, neurospheres and culture-conditioned medium (for the concentration and isolation of EVs) were collected.

### EV separation and concentration

An established ultracentrifugation protocol (Théry et al., 2006), with additional filtration steps, was used to separate and concentrate EV fractions [59], as we have previously published [32] (Fig. 1B). Briefly, cell fractions were separated from culture-conditioned medium by centrifugation at 200×*g* for 5 min, and the cell pellet isolated for separate RNA analysis. Next, the culture media supernatant was centrifuged at 2000×*g* at 4 °C for 10 min to eliminate debris and dead cells, then passed through a 0.2 µm sterile polyethersulfone membrane filter (VWR; Catalog # 28,145–501; Radnor, PA/USA) to exclude debris with diameters greater than 0.2 µm. The filtered supernatant was centrifuged through a 100 kDa molecular weight cutoff (MWCO) polyethersulfone membrane (PALL; Catalog # MAP100C37; Port Washington, NY/USA) at 4000×*g* for 30 min to concentrate the EV-enriched supernatant while filtering out any particles below 100 kDa. The collected material on the membrane was transferred to polypropylene thick-walled centrifuge tubes (Beckman; Catalog # 355,640; Brea, CA/USA), adding chilled (4 °C) 1 × PBS buffer (Thermo Fisher; Catalog # 14,190,144; Waltham, MA/USA) to a total volume of 7 mL per tube. This PBS-suspended material was centrifuged at 100,000×*g* for 90 min at 4 °C in a Type 70 Ti fixed-angle titanium rotor (Beckman; Catalog # 337,922; Brea, CA/USA). EV-enriched pellets were washed, by decanting the supernatant and resuspending the pellet in 1 mL chilled PBS, followed by the addition of 6 mL chilled PBS for centrifugation at 100,000×*g* for 90 min at 4 °C.

### Nanoparticle tracking analysis

As we have previously published [32], the size and concentration of EVs were measured by nanoparticle tracking analysis (Nanosight LM10; Malvern Panalytical; Westborough, MA/USA). Isolated EV samples were diluted, using 1 × PBS buffer (Gibco®, Thermo Fisher; Catalog # 14190144).

### Fluorescent labeling of EVs and delivery of EVs to NSCs

To visualize the uptake of EVs into NSCs in vitro, we labeled purified EVs with membrane-localized fluorochrome (PKH26, Sigma-Aldrich; Catalog # MINI26; St. Louis, MO/USA) according to the manufacturer’s instructions. Briefly, the volume of isolated EVs was brought up to 1 mL using Diluent C from the PKH26 kit for each sample, then mixed continuously for 30 s by gentle pipetting with 6 µl of PKH26 dye, then incubated in the dark, at room temperature, for 5 min. The manufacturer’s recommended quench using 10% bovine serum albumin was excluded as it resulted in aggregation of the unbound dye. PBS (Thermo Fisher; Catalog # 14190144) was added to the EV/dye mixture to a total volume of 15 mL volume and passed through a 0.2 µm sterile filter with polyethersulfone membrane (VWR; Catalog # 28145-501) to exclude possible aggregates with diameters greater than 200 nm. The filtered supernatant containing particles < 200 nm was subjected to centrifugation through a 100 kDa MWCO polyethersulfone membrane filter tube (PALL; Catalog # MAP100C37) at 4000×*g* for 30 min to concentrate the small EV-enriched supernatant while filtering out unbound dye. PBS (15 mL) was added to the EV-enriched supernatant and centrifuged once more in a 100 kDa MWCO membrane filter tube at 4000×*g* for 30 min. For every centrifugation with 100 kDa MWCO membrane filter tube, 100–200 µL of supernatant remained. The labeled EVs were rinsed an additional two times with 10 mL total volume of fresh culture media by centrifugation with 100 kDa MWCO filter tubes at 4000×*g* for 30 min, then resuspended with fresh culture media for a total volume of 1 mL. Finally, ~ 10,000 naïve NSCs were exposed to 1 mL of labeled EVs for 24 h before the cells were processed for flow cytometry or confocal fluorescence microscopy analysis.

As a negative control, fresh culture media was processed for labeling using the same procedure as that used for EVs, and then introduced to naïve NSCs to quantify any non-specific residual labeling. Cells were also directly labeled as a positive control, for which ~ 500,000 NSCs were resuspended in 0.5 mL of Diluent C and 2 µL of PKH26 dye, diluted in an additional 0.5 mL of Diluent C, was added. NSCs were incubated with dye for 5 min in the dark at room temperature with periodic mixing. To eliminate unbound dye, 1 mL of 1% BSA was added and incubated for 1 min to allow binding of excess dye. Cells were then rinsed three times with 10 mL of fresh culture media and centrifuged at 300×*g* for five minutes. Finally, direct PKH26-labeled cells were processed for flow cytometry or confocal fluorescence microscopy analysis.

### Flow cytometry

Following addition of labeled EVs, filtered culture media, or dye, recipient NSCs were briefly fixed (2% PFA, 15 min) before undergoing flow cytometry using the BD LSR Fortessa X-20 Cell Analyzer. Data were analyzed using FCS Express software v.7.12.0007 (De Novo Software).

### Confocal imaging of neurospheres

Following addition of labeled EVs, filtered culture media, or dye, cells were briefly fixed (2% PFA, 15 min). NSC nuclei were counterstained with 300 nM DAPI (Thermo Fisher; Catalog # D1306), then mounted onto glass slides (Vectashield, Vector Laboratories; Catalog # H-1200-10; Burlingame, CA/USA), coverslipped, and imaged using a confocal-laser scanning microscope (FluovView-1200, Olympus Corporation of the Americas; Center Valley, PA/USA), using a 405-nm laser to excite DAPI and a 559-nm laser to excite PKH26. Micrographs were acquired using a 60× magnification objective (UPlanSApo 60× Oil, Olympus), with additional 2× zoom through image spatial resolution adjustment.

### RNA isolation, RNA-seq library preparation, and sequencing

Total RNA from the ultracentrifuged EV pellets was isolated using the miRNeasy micro kit (Qiagen; Catalog # 217084; Germantown, MD/USA) and total RNA from the matched cell-of-origin NSC pellets was isolated using the miRNeasy mini kit (Qiagen; Catalog # 217004). RNA samples were further processed at Texas A&M Institute for Genome Sciences and Society (TIGSS) Molecular Genomics Core. Prior to analysis, RNA quality was assessed using an Agilent 2200 TapeStation RNA assay (Agilent Technologies; Catalog # G2964-90003 Rev. C; Santa Clara, CA/USA). For cell-of-origin RNA, RIN (RNA Integrity Number) values of > 9 were obtained following total RNA isolation. However, as previously documented in the literature [60–62], low levels of 28S and 18S RNA in EVs preclude accurate calculation of RIN for RNA derived from EVs (Additional file 1: Figure S1A). Total RNA concentration was quantified by High Sensitivity RNA Qubit Fluorometric Assay (Thermo Fisher; Catalog # Q32852), and all samples were adjusted to an equivalent starting concentration. Preliminary studies (*n* = 5 EV samples) with libraries prepared from total EV RNA without ribosomal RNA depletion, resulted in the majority of the mapped reads (85% – 90%) being ribosomal RNAs (Additional file 1: Figure S1B). Therefore, to increase the proportion of reads mapping to non-ribosomal RNA transcripts, sequencing libraries were prepared using SEQuoia Complete Stranded RNA Library Prep kit (Bio-Rad; Catalog # 17005726; Hercules, CA/USA) with Ribo‐Zero Plus ribosomal RNA depletion kit (Illumina; Catalog # 20040526; San Diego, CA/USA). Each sample was uniquely indexed (barcoded), then all samples were pooled for a single sequencing run. Library size and quality were then assessed using the Agilent D5000 Tape Assay (Agilent Technologies; Catalog # 5067–5588) and quantified with the High Sensitivity dsDNA Qubit assay (Thermo Fisher; Catalog # Q33230). Samples were adjusted to 4 nM and pooled equally. Sequencing was performed on Illumina NovaSeq6000 paired‐end (2 × 151) sequencing run to generate approximately 55 million read pairs per sample. Raw and processed data files are deposited in NCBI repository under accession number GSE214545.

### Bioinformatic and statistical analyses

Using the web-based Bio-Rad SeqSense analysis software pipeline (https://seqsense.bio-rad.com/), all reads for each sample were trimmed of all adapter sequences, then demultiplexed and deduplicated. As we previously published [63], analyses were conducted using the Galaxy software suite implemented on the TAMU High Performance Research Computing cluster (https://hprcgalaxy.tamu.edu/). Reads from the output *fastq* files were further trimmed of low-quality bases using *Trimmomatic* read trimmer software version 0.38.1 [64]. Using *Trimmomatic* and corresponding adapter sequence files from Illumina, reads were eliminated if the read length was < 15. Reads were then scanned with a sliding window of 4, cutting when the average quality per base drops below 25, then trimming reads at the start and end of a read if base quality drops below 25. The retained reads were mapped to the Mus musculus (mm39) genome assembly. Read mapping was performed using RNA STAR genomic analysis software platform version 2.7.8 [65]. Transcript-wise counts were calculated using HTSeq software version 0.9.1 [66]. Following the guidelines recommended by Love et al. [67], differential gene expression tests were then performed using DESeq2 software version 2.11.40.6 with an experimental design of ‘*Location’* (EV vs. Cell) × ‘*Sex’* (Female vs. Male) × ‘*Alcohol’* (0 vs. 120 vs. 320 mg/dL). Using the regularized logs of normalized gene counts derived from DESeq2, a total of 40,205 genes had at least one read count in at least one sample and were processed for differential expression analysis.

Data from the differential gene expression tests were used to construct volcano plots using the *EnhancedVolcano* R package [68]. Pathway analysis was conducted on differentially expressed genes using *ReactomePA* R package [69] which utilizes the KEGG database [70] and the *Pathview* R package [71] to visualize differentially regulated pathways. Following the guidelines recommended by Langfelder et al. [72], weighted gene co-expression network analysis (WGCNA) was conducted using *WGCNA* R package [72] to identify clusters/modules of genes with highly correlated expression levels, to measure the relationships between modules and sample traits, to identify hub genes within modules that have significantly strong correlations with sample traits of interest (location to indicate cell-of-origin cell or EV, pregnancy as the biological replicate, and sex), and to perform gene ontology enrichment analysis on the identified modules. Briefly, the Pearson correlation coefficient was calculated to assess the similarity of the gene expression profiles. Then, correlation matrices were converted into adjacency matrices, which were then weighted by a soft power function (14 for WGCNA of all 36 samples; 9 for independent WGCNA of EV samples and cell samples; 6 for *Consensus* WGCNA) to obtain a scale-free network. The adjacency matrix was converted into a topology overlap matrix (TOM), and TOM was used as input to hierarchical clustering (network type = signed), with modules (*minModuleSize* set at 30) detected by cluster analysis during module selection. Each module was represented by a unique color code label. The ‘merged tree-cut’ method was used to identify different modules, with a cut height for merging of modules at 0.20 (i.e., modules whose eigengenes are correlated above *r* = 0.8 were merged into a single module) for the WGCNA of all 36 samples and additional independent WGCNA of EV samples and cell samples, and with the cut height for merging of modules at 0.25 for consensus WGCNA. Consensus WGCNA examined all 36 samples as two individual data sets of EV samples and cell samples, and identified modules/clusters of genes that are highly interconnected in both sets. After modules are identified, the relationships between modules and sample traits were measured by calculating the Pearson correlation coefficient between modules and sample traits. As the representative gene expression profile of the module, each module eigengene (ME) was correlated to sample traits of interest, resulting in both correlation and p values of MEs to each trait of interest. After finding the sample trait that correlated with most modules, gene significance (GS) of each gene to that sample trait was measured. Here, GS was defined as the absolute value of the correlation between the trait and a gene’s expression profile, with Student asymptotic *p*-value for given correlations, and used to assess the biological significance of a given gene to a sample trait. A higher GS value indicates that the gene is a better predictor of the sample trait [72]. This is useful since multiple genes, and not just the hub gene, with high GS in a module can be studied as top candidates in relation to a sample trait of interest. Module significance (MS) was calculated as the average GS of all genes in a module, to consider modules with the highest MS as the most relevant modules to the sample trait of interest. Therefore, the correlation of MEs to sample traits helped to choose which sample trait to use for GS, and MS was calculated to observe modules that may be most biologically relevant modules to the sample trait of interest. This dual approach, in examining the relationship between modules and sample traits, shows how each module relates to various sample traits to find the most relevant modules and genes to the sample trait of interest.

Preliminary principal component analysis showed that RNA transcripts that contributed to both the first and second principal components separated samples by pregnancy (Additional file 2: Figure S2), suggesting that RNA content of cells and EVs can vary substantially from one pregnancy to the next. Therefore, to reduce the contribution of pregnancy and litter to variability in gene expression, we used a repeated-measures design for parametric testing, with pregnancy/litter identity as a within-subjects measure for both sex and ethanol treatment. To identify RNA transcripts where EV enrichment was significantly altered by treatment, we used three criteria, a paired samples t-test, a Hedges’ g (‘g’) effect size, and a 95% confidence estimate for the effect size using the *Effsize* R package, v.0.8.1, to compare the means between control and treatment groups. For pathway analysis, RNA transcripts were selected when their EV-to-cell enrichment was altered by treatment with a *p*-value < 0.05, Hedges’ *g* > 0.4, and with 95% confidence estimate for Hedges’ *g* that was non-zero containing. Enrichment pathway analysis was conducted using *ReactomePA* R package [69] and *ClusterProfiler* R package [73]. All other statistical analyses were conducted using the “R” software (R Foundation for Statistical Computing; Vienna/Austria), v.4.2.1 for Windows, “RStudio” software (RStudio, Inc.; Boston, MA/USA), v.2022.07.1 for Windows, and the “GraphPad Prism” software (GraphPad Software; San Diego, CA/USA), v.9.4.1 for Windows. The ‘R’ code generated by DDC for this study is available on GitHub (https://github.com/daehyukchung/EV_Cell_Transcriptomic.git). All sample sizes, statistical tests, and post hoc analyses are appropriately reported in “Results” section.

### Data analysis of scRNA sequencing

We analyzed our previously published scRNAseq data from GD 14.5 mouse cerebral cortex (NCBI GEO accession number GSE158747) [13], for which fetal mice were exposed to vaporized ethanol or room air on GD 12.5, to validate fetal NSCs as the source of ethanol-sensitive RNA transcripts secreted in EVs. We focused on cell clusters that we previously identified as ventricular zone (VZ), subventricular zone (SVZ), cells transitioning from VZ to SVZ (VZ/SVZ), and transit progenitor cells (TPC), which are the cell populations of the developing fetal cortex that are most closely modeled in our neurosphere cultures [13]. After re-clustering cell populations with VZ, SVZ, VZ/SVZ, and TPC identity, as previously published [33], the cell-types expressing the composite transcriptomic signature of the differentially regulated genes in EVs (*p* < 0.05; Hedges’ *g* > 0.4 with a non-zero containing 95% confidence estimate), from both the 120 and 320 mg/dL exposure conditions, were identified.

## Results

### Fluorescence-labeled EV uptake by NSCs

**Fig. 2:**
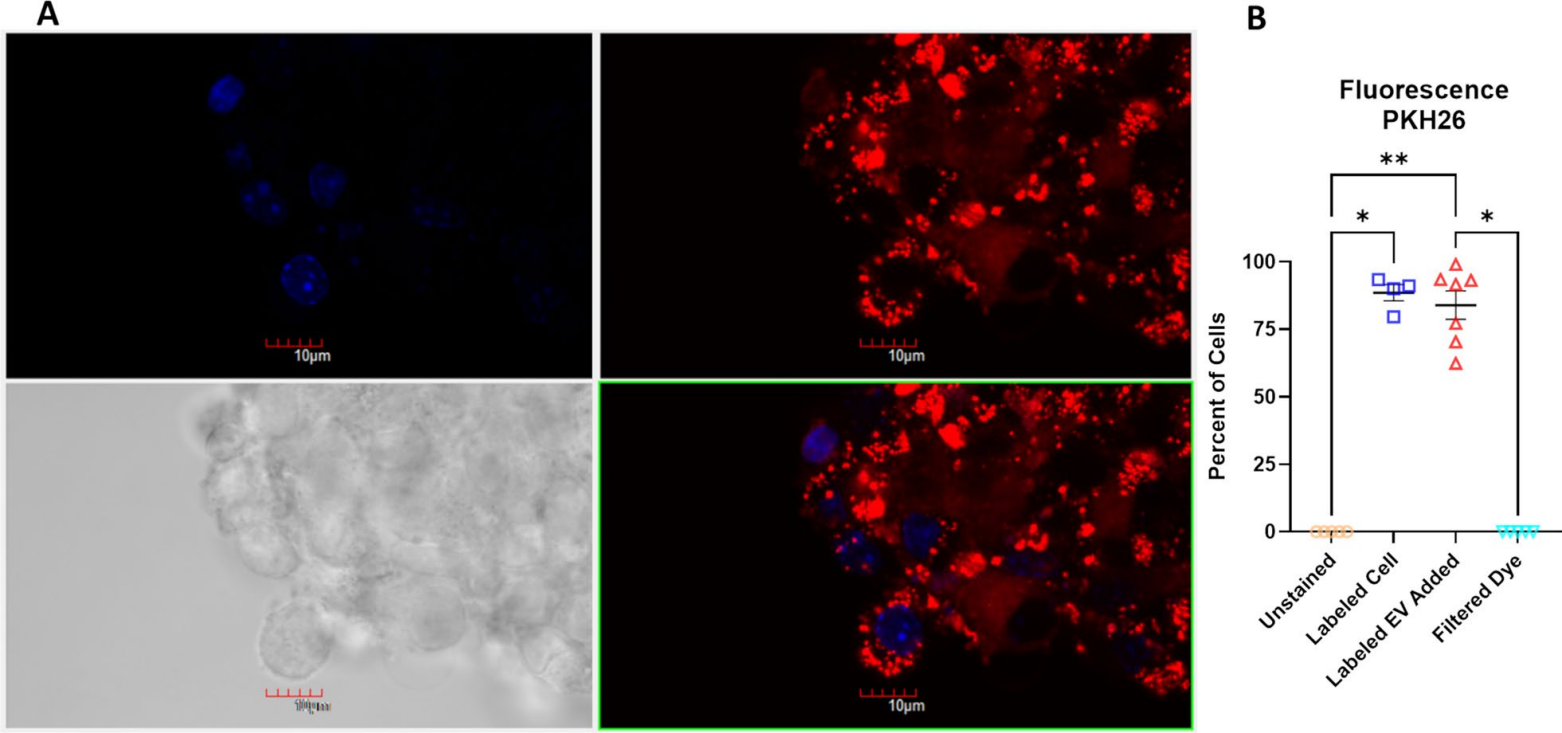
Fluorescent-Labeled EV Uptake by Cells. **A** Confocal fluorescent microscopy image of PKH26-labeled EVs taken up by naïve NSCs; PKH26-labeled EVs are red and NSC nuclei counterstained with DAPI are blue. **B** Flow cytometric analysis for proportion of label-positive NSCs in unstained control cells, cells directly labeled with PKH26, cells that have sequestered labeled EVs, and cells that were incubated with purified culture medium spiked with PKH26 dye, that were then processed for the same labeling and filtration protocol as that used for isolated EVs; *n* = 4 to 7 samples per group; Kruskal–Wallis test for nonparametric one-way ANOVA, approximate *p*-value < 0.0001 followed by Dunn’s multiple comparisons post hoc test, *p < 0.05, **p < 0.01

We defined EVs by their molecular composition and size, as we have published previously [32]. EVs were isolated from either female or male mouse fetal NSC cultures obtained from three separate pregnancies, and from control or ethanol-exposed conditions and purified as previously described (Fig. 1A, B). Nanoparticle tracking analysis (NTA) showed that, after 48 h of incubation with ~ 10^6^ NSCs, culture-conditioned media contained ~ 10^9^ EVs that ranged from 50 to 200 nm in diameter with 150 nm as the median diameter (Fig. 1C), consistent with the known size range for small EVs and exosomes [28, 74]. Purified EVs, labeled with PKH26 fluorescence reporter, were taken up by ethanol-naïve NSCs and were detected within the cytoplasm of recipient NSCs (Fig. 2A). Flow cytometric analysis of labeled-EV-positive NSCs indicated that ~ 84% of naïve NSCs incorporated labeled EVs following incubation (Fig. 2B), indicating robust EV uptake in our neurosphere cultures. Moreover, this labeling can be attributed to the uptake of EVs, while NSCs could be directly labeled with PKH26 (Additional file 3: Figure S3), as culture media that was mixed with PKH26 and then processed through the same purification steps used for labeled EVs did not result in labeling of recipient NSCs (Additional file 4: Figure S4).

## Transcriptome profile of EVs differs from their cell-of-origin NSCs

**Fig. 3:**
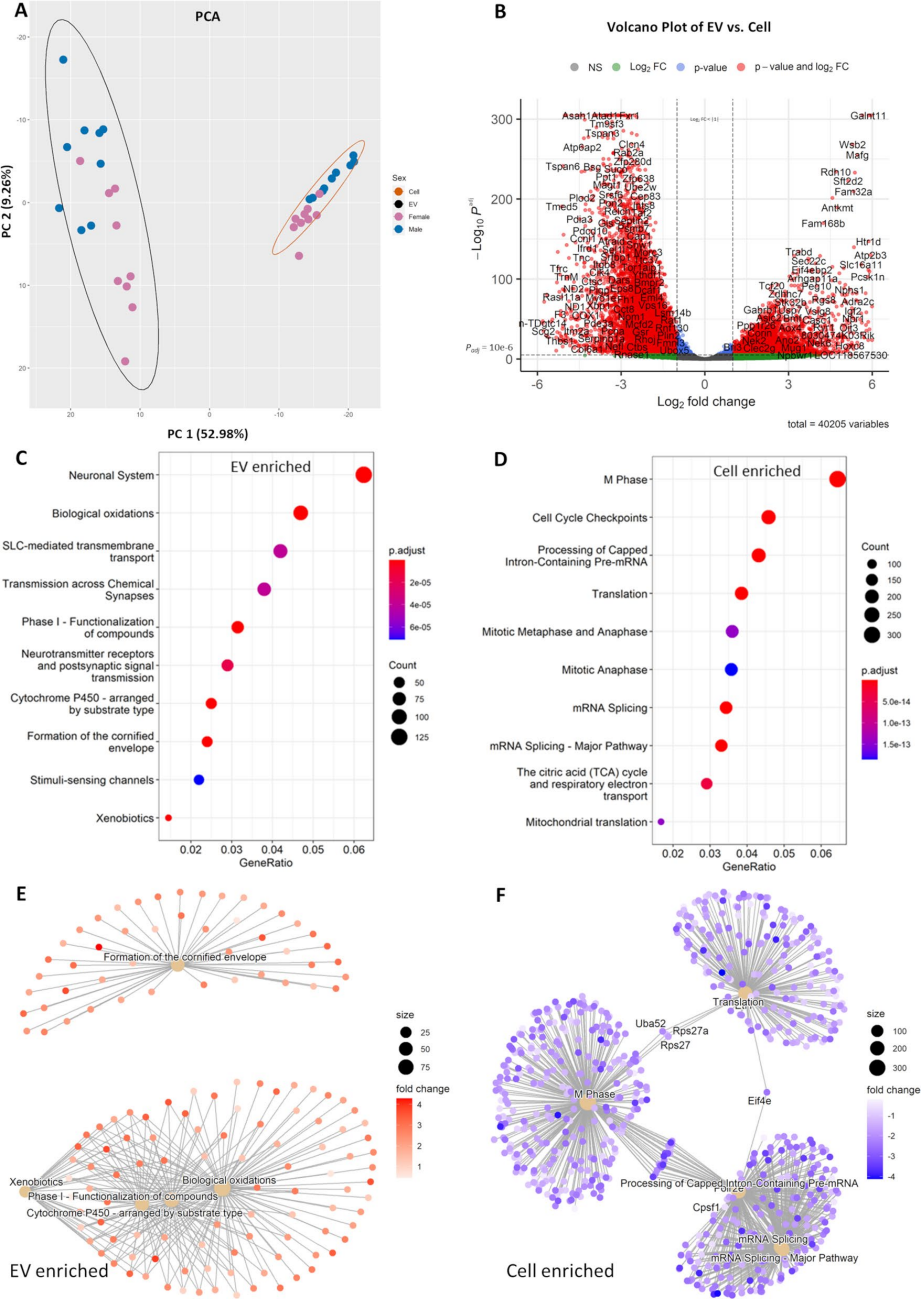
Differential Gene Expression and Pathway Overrepresentation Analysis. **A** Principal component analysis was performed on the 500 most variant RNA transcripts from 18 NSC parental cell samples and their corresponding 18 EV samples. This analysis shows that the samples could be segregated with the 1^st^ principal component, by the sample trait of ‘*Location*’ (whether the RNA transcripts were from EV or cell sample). **B** Volcano plot of log2 fold change and − log10 *p*-value of all genes differentially expressed in EV samples vs. their parental cell samples (EV, *n* = 18; Cell *n* = 18). **C** Dot plot depicting pathway overrepresentation (based on *ReactomePA)* for significantly altered genes (adjusted *p* < 0.05) enriched in EV samples relative to their parental cell samples. **D** Dot plot depicting pathways related to significantly altered genes (adjusted *p* < 0.05) enriched in cell samples relative to their derived EV samples. Each plot presents overrepresented pathways, ordered by gene ratio, i.e., the proportion of differentially expressed genes/transcripts within an ontology term. The size of each dot denotes the number of genes/transcripts in a pathway that were contained within this dataset, while the color of each dot encodes the Benjamini and Hochberg-adjusted p-value for significance of pathway overrepresentation. *n* = 18 EV samples, 18 cell samples. **E**, **F** Graphical representation of the relationship between enriched pathways and their associated genes/transcripts. Significantly altered genes (adjusted *p* < 0.05) were selected for this analysis. Pathways that reached a Benjamini–Hochberg false discovery rate-adjusted *p* value criterion of < 0.05 were selected. The size of each filled central circle represents the number of transcripts in a pathway that were overexpressed in EVs (**E**) or in cells (**F**). The color of each dot associated with that pathway denotes the fold change for that transcript in EVs relative to parental cells

All RNAseq data used for these analyses have been deposited in NCBI Gene Expression Omnibus, under accession number GSE214545. Whole-transcriptome RNA sequencing (RNA-seq) analysis of ribosomal RNA-depleted EV and cell-of-origin NSC RNA identified 40,205 unique genes present in at least one of the 36 assessed samples (18 EV and 18 corresponding cell-of-origin NSC samples, Additional file 7: Table S1). Principal component analysis of the 500 most variant RNA transcripts showed that PC1 (52.98% of total variance) segregated samples based on sample source, i.e., EV vs. cell, and PC2 (9.26% of sample variance) segregated samples by sex, i.e., female vs. male EVs and female vs. male cell-of-origin NSCs (Fig. 3A; Additional file 5: Figure S5; Additional file 8: Table S2). From these 500 most variant genes, 128 were significant differentially expressed genes (DEGs) by sex in EVs compared to 102 significant DEGs by sex in cells. Among these DEGs, 31 were shared by EVs and cells, therefore ~ 76% of sex-associated DEGs in EVs were unique to EVs and were not significant DEGs in cells-of-origin.

To assess the effect of sample source (EV vs. cell) on transcript expression, we analyzed the distribution of transcript enrichment. RNA‐seq analysis identified 21,539 significantly differentially expressed genes (DEGs; FDR‐corrected *p* < 0.05), including 10,422 downregulated genes (48.39%) and 11,117 enriched genes (51.61%) in EVs compared to cells (Fig. 3B; Additional file 3: Figure S3).

We next examined potential biological functions of DEGs enriched in EVs or in cells through pathway analysis (adjusted *p* < 0.05) to identify significant biological pathways for the DEGs (Fig. 3C, D; Additional file 10: Table S4, Additional file 11: Table S5). For EV-enriched DEGs, pathways associated with intercellular transport and transmission were overrepresented (Fig. 3C; Additional file 10: Table S4). For cell-enriched DEGs, pathways associated with broad cellular processes, such as translation and cell cycle, were overrepresented (Fig. 3D; Additional file 11: Table S5). Additional network analyses (Fig. 3E, F; Additional file 10: Table S4, Additional file 11: Table S5) show the contribution of individual genes to core overrepresented pathways. Overall, our data show a preferential transfer of distinctly different classes of genes, that are a part of largely non-overlapping biological pathways, from cell-of-origin NSCs to their secreted EVs.

### Weighted gene co-expression network analysis to compare the transcriptome of EVs to cells

Weighted gene co-expression network analysis (WGCNA) was used as a systems biology approach to: (a) assess the interconnection between genes and find biologically significant clusters of genes (modules); (b) identify modules with highly correlated expression levels and hub genes within each module; (c) measure the relationships between modules and sample phenotypic traits; (d) determine hub genes that have significant correlations with phenotypic traits of interest; and (e) analyze identified modules for gene ontology enrichment analysis to determine potential biological relevance of these gene clusters.

**Fig. 4:**
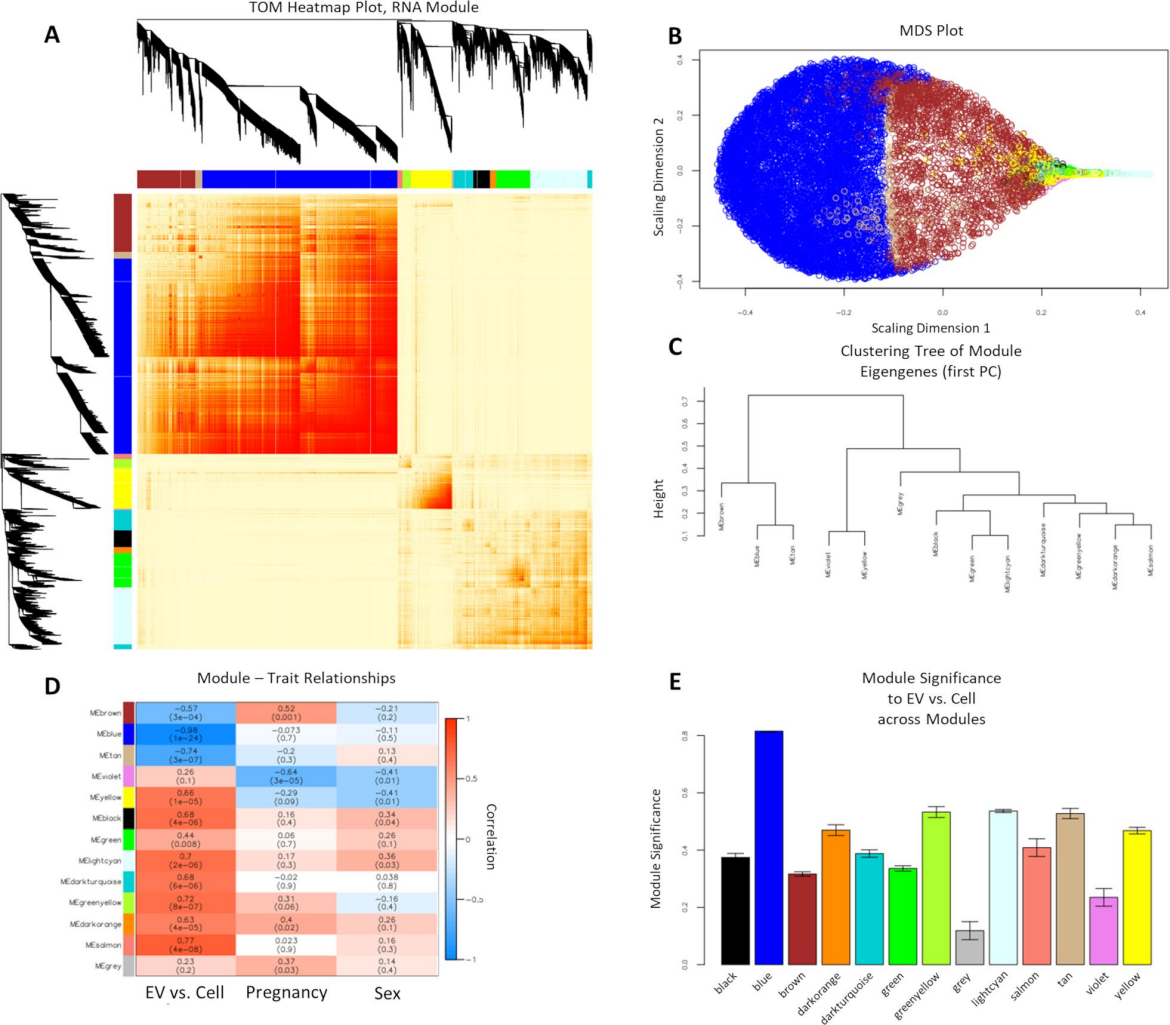
WCGNA-based Comparisons of the RNA Content of EVs Relative to Parental NSCs. WGCNA of RNA transcripts expressed in all 36 samples, shows that EVs contain a distinct set of gene networks compared to and their parent NSCs. Correlations with the trait of ‘*Location’* (EV vs. cell) contributed to the majority of the difference between clusters. Other traits including ‘*Pregnancy*’ (NSCs derived from three separate pregnancies) and ‘*Sex’* (female or male fetal sex) were smaller, but also significant contributors to the overall composition of identified networks. **A** Topological overlap matrix (TOM) plot for visualizing the weighted gene co-expression network, where the topological overlap considers each pair of genes’ similarity in relation to all other genes in the network. Modules are defined by hierarchical clustering, visualized by dendrograms and module color notation. Each row or column corresponds to a single gene, where lighter red denotes low topological overlap and darker red denotes high topological overlap. The *grey* module represents residual genes that could not be associated with networks. **B** Multidimensional Scaling (MDS) plot where each point represents a gene transcript. The distances between any two pair of points is a measure of the dissimilarity between those two genes. As visualized, blue and tan and brown module transcripts (enriched in cells) segregate from each other, and also from all other modules (EV-enriched modules). **C** Module eigengene (ME) dendrogram as a measurement of dissimilarity between MEs (1st principal component), showing that ME brown, ME blue, and ME tan are separated from the rest of the MEs. **D** Relationship between WGCNA modules and external sample traits of ‘*Location*’, ‘*Pregnancy*’ and ‘*Sex*’. Each row depicts a module eigengene (ME), identified by a unique module color identity, with sample traits arranged across columns. Numbers in the table correspond to the correlation coefficients between the ME and the specific trait, with p-value in parentheses. The strength and direction of the correlation is illustrated by the color legend, with red depicting positive and blue depicting negative correlation with a numerically encoded trait. The color intensity indicates the strength of the correlation. This analysis shows that ‘*Location’* (EV vs. cell) is the most important determinant of module identity, with the brown, blue, and tan modules identifying cells, and the majority of the other modules identifying EVs. However, some modules also exhibit moderate but statistically significant correlations with the traits of ‘Pregnancy’ and ‘Sex’. **E** Bar plot of ‘*Location’* (EV vs. cell) trait-based module significance (average gene significance in a module) across modules. Gene Significance (GS) is a measure biologically significant a given gene is to a sample trait, and module significance is calculated from the average gene significance of all genes in a module in relation to a phenotypic trait of ‘*Location’*

Firstly, WGCNA was performed on all 36 samples (18 EV and 18 cell-of-origin NSC samples). This WGCNA identified 13 modules of correlated genes that were assigned unique color codes, including ‘*grey*’ for a module of genes that did not correlate well with genes in any of the other 12 modules (Additional file 12: Table S6). Hub genes within each module were also identified (Additional file 13: Table S7). We used a topological overlap matrix (TOM) heatmap plot (Fig. 4A) and a multidimensional scaling (MDS) plot (Fig. 4B) as graphical representations of network connection strength within and between modules. We found that the *blue*, *tan*, and *brown* modules were highly intercorrelated, but poorly correlated with the expression of genes in all other modules. This outcome implies that genes from the three (*blue*, *tan*, and *brown*) modules have similar expression patterns, with many shared networks. This relationship between modules was further seen using a module eigengene (ME; first principal component of a given module) dendrogram in which the *blue*, *tan*, and *brown* modules were in one group on the first major branch and the other modules all were in the other major branch (Fig. 4C).

To assess the relationship between each module and three sample traits, ‘*Location’* (i.e., EV vs. cell), ‘*Pregnancy’* (i.e., independent biological replicates which were denoted as ‘set’ numbers), and ‘*Sex’* (i.e., female vs. male), we identified modules that are significantly associated with sample traits by correlating MEs with traits (Fig. 4D). We observed that there was a significant correlation between a majority of MEs and the sample trait of ‘*Location’* (EV vs. Cell), with eigengenes of *blue* (*r* =  − 0.98, *p* = 1×10^−24^), *tan* (*r* =  − 0.74, *p* = 3×10^−07^), and *brown* (*r* =  − 0.57, *p* = 3×10^−4^) modules having significant inverse correlations with ‘*Location’* (i.e., higher in cell-of-origin NSCs relative to EVs), while the majority of the remaining module eigengenes had significant positive correlations with ‘*Location’* (i.e., higher in EVs relative to cell-of-origin NSCs), with eigengene of *salmon* (*r* = 0.77, *p* = 4×10^−08^) module having the highest significant positive correlation, followed by eigengenes of *greenyellow* (*r* = 0.72, *p* = 8X10^−07^) and *lightcyan* (*r* = 0.7, *p* = 2×10^−06^) modules. For sample trait ‘*Pregnancy’*, eigengenes of *violet* (*r* =  − 0.64, *p* = 3×10^−05^), *brown* (*r* = 0.52, *p* = 0.001), and *darkorange* (*r* = 0.4, *p* = 0.02) modules had significant correlations, suggesting that these MEs varied significantly from one pregnancy to another. For sample trait ‘*Sex’*, eigengenes of *violet* (*r* =  − 0.41, *p* = 0.01) and *yellow* (*r* =  − 0.41, *p* = 0.01) modules had a significant inverse correlation (i.e., higher in males relative to females), while eigengenes for *black* (*r* = 0.34, *p* = 0.04) and *lightcyan* (*r* = 0.36, *p* = 0.03) modules had significant positive correlations (i.e., higher in females relative to males).

Since a majority of the WGCNA-defined modules significantly correlated with sample trait ‘*Location’*, we examined the module significance (average absolute gene significance) across modules in relation to the sample trait ‘*Location’* (Fig. 4E; Additional file 13: Table S7). By taking the average GS of all genes in a module in relation to a sample trait, ‘*Location’* (EV vs. cell) in this case (Additional file 14: Table S8), we can better understand the significance of each module to this sample trait. For sample trait ‘*Location’*, the *blue* module had the highest module significance value of 0.82 (Module Gene Count: 8477; Hub Gene: *Kpna1*), followed by *lightcyan* module (Module Significance: 0.54; Module Gene Count: 8477; Hub Gene: *Ush2a*), *greenyellow* module (Module Significance: 0.53; Module Gene Count: 377; Hub Gene: *Casc1*), and *tan* module (Module Significance: 0.52; Module Gene Count: 298; Hub Gene: *n-TLtag3*) (Fig. 4E, Additional file 13: Table S7), indicating that these modules were the best predictors of ‘*Location*’.

Next, we performed gene ontology enrichment analysis on the identified modules (Additional file 15: Table S9) for modules with high module significance and high correlation to sample trait ‘*Location’*, whose hub gene was also significantly differentially expressed in EVs compared to cells as determined by the DESeq2 analysis of the distribution of transcript enrichment (Additional file 9: Table S3). The *blue* module exhibited the highest module significance value (0.82) (Additional file 13: Table S7) and the highest correlation (*r* =  − 0.98) (Fig. 4D) with the sample trait ‘Location’. The *blue* module hub gene, *Kpna1*, was significantly enriched in cells compared to EVs (log_2_(fold change) =  − 2.20; adjusted *p* = 2.25 × 10^–117^) (Additional file 9: Table S3). Gene ontology of the *blue* module revealed that genes within this network are most associated with cellular macromolecule biosynthetic process and regulation of synapse organization, structure, or activity (Additional file 15: Table S9) and *Kpna1*, karyopherin (importin) alpha 1, is predicted to enable nuclear protein import (Gene/Entrez ID 16,646). The *lightcyan* module had the highest module significance (0.54) (Additional file 13: Table S7) for a module with a positive correlation (*r* = 0.7) (Fig. 4D) to sample trait ‘*Location’* (i.e., higher in EVs relative to cell-of-origin NSCs), and hub gene *Ush2a* was significantly enriched in EVs compared to cells (log_2_(fold change) = 2.03; adjusted *p* = 7.86 × 10^–41^) (Additional file 9: Table S3). Gene ontology of the *lightcyan* module revealed that genes within this network were involved in chaperone binding and protein sequestering activity (Additional file 15: Table S9) and hub gene *Ush2a*, usherin, encodes a basement membrane associate protein that contains laminin EGF motifs, a pentaxin domain, and many fibronectin type III motifs (Gene/Entrez ID 22283).

### Consensus WGCNA for shared and divergent modules between EVs and cells

**Fig. 5:**
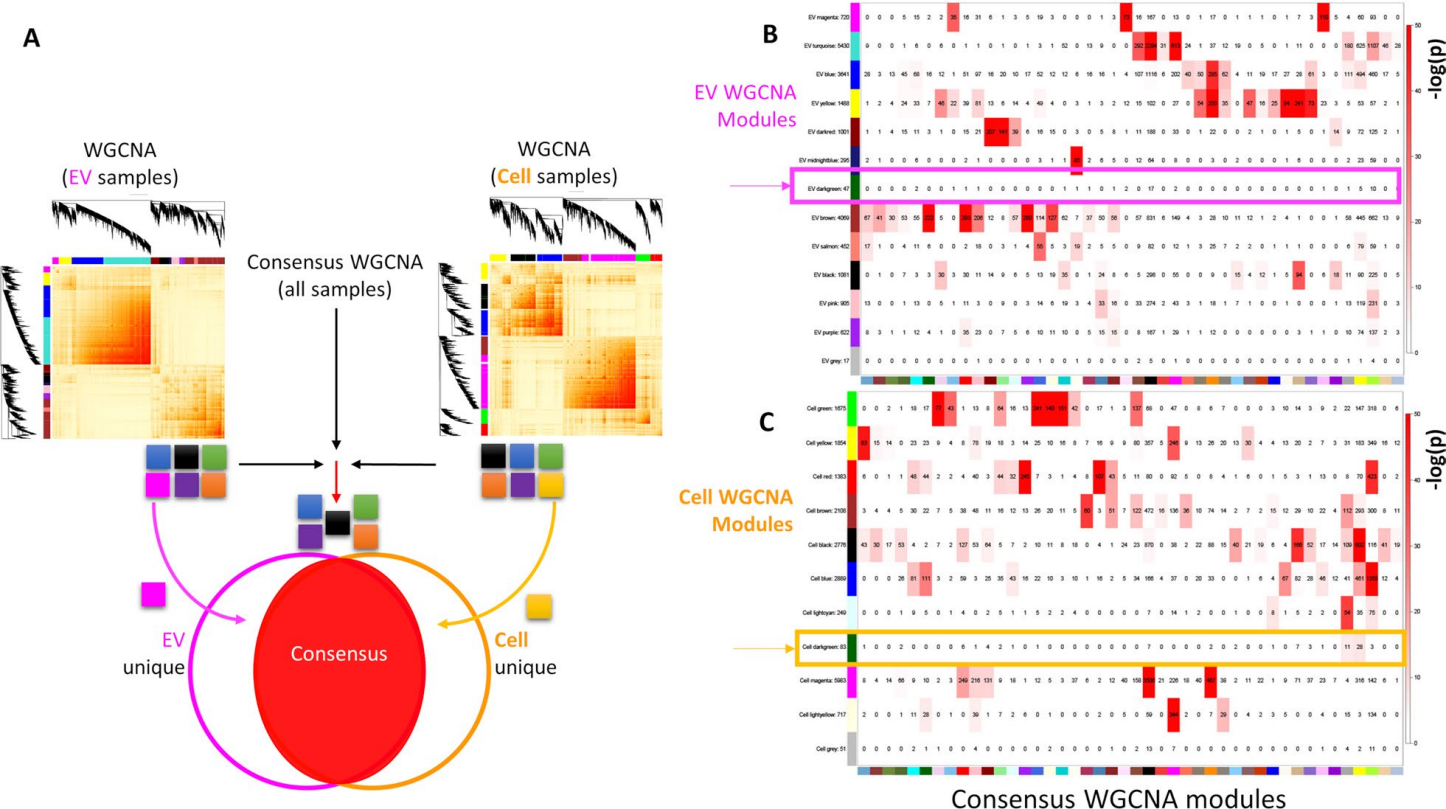
WCGNA-based Comparisons of the Consensus Modules to EV WGCNA-identified and Cell WGCNA-identified Modules. **A** Schematic diagram for the consensus WGCNA process. **B** Color-coded correspondence table of EV sample WGCNA-identified modules and the EV-Cell consensus modules indicates that most EV sample WGCNA-identified modules have a consensus counterpart, except for EV ‘*darkgreen*’ module (magenta box with arrow) which is unique to EVs. **C** Color-coded correspondence table of Cell sample WGCNA-identified modules and the EV-Cell Consensus modules indicates that most cell sample WGCNA-identified modules have a consensus counterpart. However, Cell ‘*darkgreen*’ (orange box with arrow, unrelated to EV *‘darkgreen’*), exhibits minor overlap with EV-Cell Consensus modules ‘*darkgrey’* and ‘*yellow’*, representing a nearly unique Cell module. Numbers in the table indicate gene counts in the intersection of the corresponding modules. Coloring of the table encodes − log(p), with p being the Fisher’s exact test *p* value for the overlap of the two modules. The *grey* module represents residual genes that could not be associated with networks

Since WGCNA on all 36 samples identified modules that were significantly associated with ‘*Location*’, we set out to further understand how the gene networks of EVs differ from the gene networks of their cell-of-origin NSCs. We therefore used a *Consensus* WGCNA methodology [75] to identify modules which were shared by EVs and Cells, known as ‘*Consensus*’ modules, and more importantly, modules which uniquely defined EVs as different from their cell-of-origin NSCs. We therefore performed two separate WGCNAs, one on the 18 EV samples and one on the 18 cell samples. WGCNA in EVs identified 12 modules of highly correlated genes and their hub genes (Fig. 5A; Additional file 16: Table S10, Additional file 17: Table S11), excluding the *grey* module for unassigned genes. For the Cell data set, WGCNA identified 10 modules, excluding the grey module for unassigned genes, their correlated genes (Additional file 18: Table S12), and their hub genes (Additional file 19: Table S13). We then performed Consensus WGCNA on all 36 samples to identify *Consensus* modules that were conserved between EV and cell data sets, and compared EV WGCNA modules and cell WGCNA modules to the consensus WGCNA modules (see Fig. 5A for schematic of protocol).

We next determined the extent to which *Consensus* modules were conserved in EV and Cell WGCNAs, by calculating the overlap of gene representation, i.e., the number of genes in the intersection of both modules, in each paired comparison between EV and *Consensus* module or Cell and *Consensus* module. Overlap significance was then determined by Fisher’s exact test to assign a p-value to each of the pairwise overlaps. The higher the percentage of genes present in both a consensus module and an EV or cell module, the more similar that consensus module is to the EV or cell module in its gene network. The majority of EV WGCNA- and Cell WGCNA-identified modules had significant overlap with at least one of the *Consensus* WGNCA modules (Fig. 5B, C). However, we also identified EV and Cell modules with little-to-no overlap with the *Consensus* modules, indicating that these modules were unique to EVs or Cells. For EV WGCNA modules, the *darkgreen* module had no *Consensus* counterpart and the *midnightblue* module only had one *Consensus* counterpart (Fig. 5B). Gene ontology of the EV *darkgreen* module identified the pathway for RNA polymerase I transcription activity (Additional file 16: Table S10, Additional file 20: Table S14). Gene ontology of the EV *midnightblue* module identified genes associated with meiotic division and cell differentiation (Additional file 16: Table S10, Additional file 20: Table S14). Likewise, for Cell set-specific modules, the cell *darkgreen* module had the lowest number of *Consensus* counterparts with two consensus modules (Fig. 5C). Gene ontology of the cell *darkgreen* module revealed genes associated with rRNA catabolic process and snRNA 3′-end process in the nucleus (Additional file 18: Table S12, Additional file 21: Table S15).

**Fig. 6:**
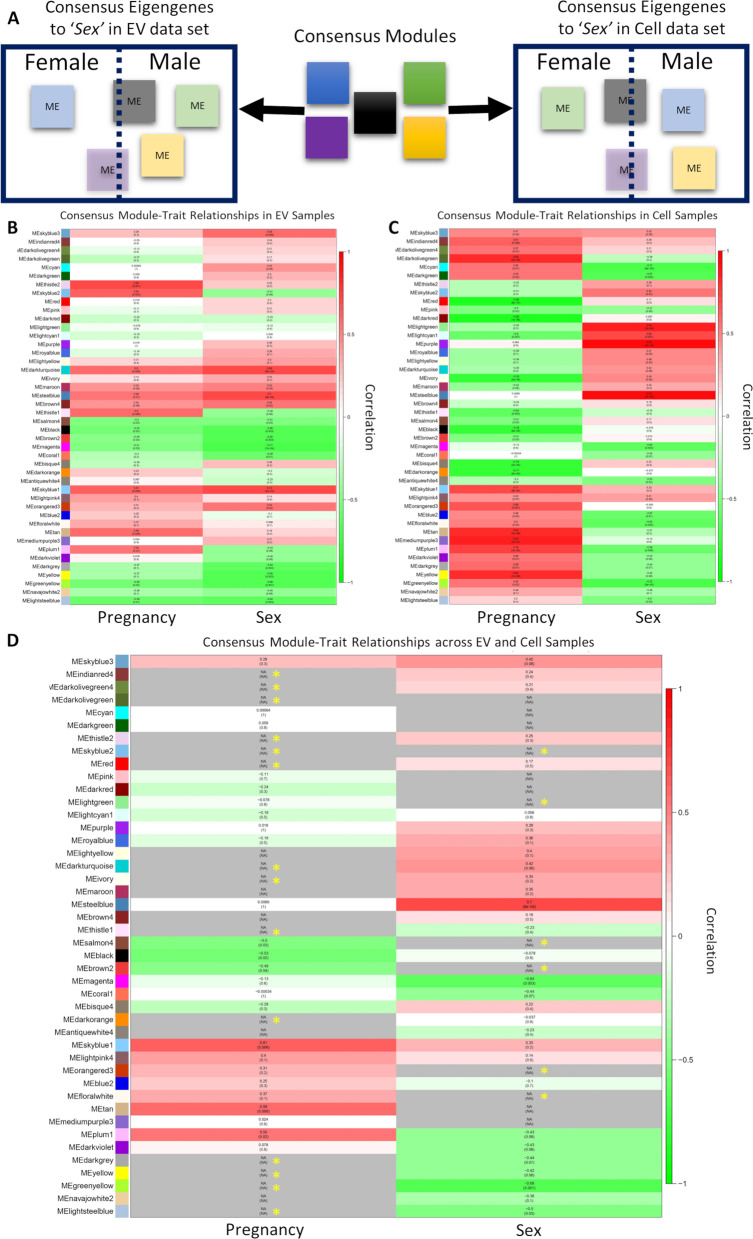
WCGNA-based Relationship between EV – Cell Consensus Modules and Sample Traits. **A** Schematic diagram of the relationship between eigengenes from consensus modules and sample trait of ‘*Sex*’ (as a trait example) in EV or Cell data sets. **B**–**D** Color-coded heatmaps of Consensus module relationship to sample traits of ‘Sex’ and Pregnancy’ in EV samples (**B**), Cell samples (**C**), or across both EV and cell samples (**D**). The rows depict Consensus module eigengenes (MEs) and their unique module color identities, while the columns depict the sample traits, ‘Sex’ and ‘Pregnancy’. Numbers in the table correspond to the correlation coefficients between the ME and the specific trait, with *p*-value in parentheses. The strength and direction of correlation is represented by a color scale, where red indicates positive and green indicates negative correlation with a trait. For ‘**D**’, consensus module–trait relationships across EV and cell samples were constructed by taking the lower absolute value of each module’s correlation coefficient from the two data sets if the two correlations have the same sign. Correlation coefficients were set to ‘NA’, and color-coded to grey, when the two correlations had opposite signs. Yellow asterisks within grey-coded cells indicate that the sample trait correlation with eigengene was significant in either Cell or EV dataset. In both EV and Cell samples, there were significant correlations between consensus modules and sample traits of ‘*Pregnancy’* and ‘*Sex’,* though the correlation was in the opposite direction for cells and EVs

We next related the sample traits of ‘*Sex*’ and ‘*Pregnancy*’ from which fetal NSCs were derived, to *Consensus* module eigengenes (*ME*s, representing the first Principal Component of a module) in each of the EV and Cell datasets. While the identity of genes assigned to a specific *Consensus* module is expected to be constant between EV and Cell datasets, i.e., a consensus, there may be sex differences, or differences from one pregnancy to the next, in the expression level of specific module genes and these differences may not be represented similarly in EVs compared to Cells. To test the hypothesis that gene expression level of a *Consensus* module was dependent on fetal sex and pregnancy*,* we generated separate EV and Cell sets of *Consensus ME*s and examined their relationships to sample traits of ‘*Sex*’ and ‘*Pregnancy*’ (Fig. 6A models the test for the trait ‘*Sex*’). The relationship between *Consensus* modules and sample traits were visualized using color-coded heatmaps of correlation values and corresponding significance (p)-values (Fig. 6; Additional file 22: Table S16). We identified 11 consensus modules that were significantly associated with sample traits of ‘*Pregnancy’* or ‘*Sex’*, for both EV and Cell samples. For the significant modules, 90.91% had similarly signed correlation coefficients (i.e., exhibited directionally similar correlation coefficients) in EV and Cell samples and 9.09% had oppositely signed correlation coefficients (i.e., modules exhibited opposite correlation coefficients in EV samples compared to cell samples) (Fig. 6B, C). For consensus modules with opposite correlation coefficients for sample trait ‘*Sex*’, this indicates that the module gene networks are overexpressed in one sex, i.e., female, in EVs relative to cells, but overexpressed in the opposite sex, i.e., male, in cells relative to EVs. To visually summarize the two sets into one measure, we constructed a third heatmap for the consensus module–trait relationships across EV and Cell samples. Here we selected the lower absolute value of each module’s correlation coefficient from the two data sets if the two correlations had the same sign, and setting the correlation coefficient to ‘NA’ to indicate zero relationship when the two correlations have opposite signs, i.e., positive in one sex, but negative in the other (Fig. 6D). Thus, 13 out of 44 consensus modules were designated as ‘NA’ meaning these consensus module’s relationship to sample trait ‘*Sex’* is dependent on the sample trait ‘*Location’* (EV vs. cell). From these 13 modules, 6 modules (Fig. 6D, asterisks) were significantly associated with sample trait ‘*Sex’* in either EV or cell samples (*skyblue2* (EV: *r* =  − 0.41, *p* = 0.09; cell: *r* = 0.55, *p* = 0.02), *lightgreen* (EV: *r* =  − 0.12, *p* = 0.4; cell: *r* = 0.84, *p* = 2e-06), *salmon4* (EV: *r* =  − 0.51, *p* = 0.03; cell: *r* = 0.17, *p* = 0.5), *brown2* (EV: *r* =  − 0.65, *p* = 0.003; cell: *r* = 0.074, *p* = 0.8), *orangered3* (EV: *r* = 0.53, *p* = 0.02; cell: *r* =  − 0.045, *p* = 0.9), and *floralwhite* (EV: *r* = 0.098, *p* = 0.7; cell: *r* =  − 0.62, *p* = 0.005)). Collectively, these data show that ~ 70% of the Consensus modules between EVs and Cells were sex-invariant and for ~ 17% of the modules, the sex-dependency of the modules was undetermined. However, for 13% of the consensus modules, the transcript abundance in EVs relative to cells within those modules was sex-dependent.

For sample trait ‘*Pregnancy’,* 19 out of 44 consensus modules were designated as 'NA', meaning these consensus modules’ relationship to sample trait ‘*Pregnancy’* was dependent on the sample trait ‘*Location’* (EV vs. cell). From these 19 modules, 14 modules (~ 31% of all modules, Fig. 6D, asterisks) were significantly associated with sample trait ‘*Pregnancy’* in either EV or cell samples (*indianred4* (EV: *r* = -0.05, *p* = 0.8; cell: *r* = 0.61, *p* = 0.006)*, darkolivegreen4* (EV: *r* =  − 0.12, *p* = 0.6; cell: *r* = 0.57, *p* = 0.01)*, darkolivegreen* (EV: *r* =  − 0.27, *p* = 0.3; cell: *r* = 0.83, *p* = 5e-06)*, thistle2* (EV: *r* = 0.69, *p* = 0.001; cell: *r* =  − 0.32, *p* = 0.2)*, skyblue2* (EV: *r* = 0.63, *p* = 0.004; cell: *r* =  − 0.31, *p* = 0.2)*, red* (EV: *r* = 0.016, *p* = 0.9; cell: *r* =  − 0.86, *p* = 8e-07)*, darkturquoise* (EV: *r* = 0.6, *p* = 0.008; cell: *r* =  − 0.35, *p* = 0.2)*, ivory* (EV: *r* = 0.14, *p* = 0.6; cell: *r* =  − 0.78 *p* = 5e-05)*, thistle1* (EV: *r* = 0.6, *p* = 0.008; cell: *r* =  − 0.64, *p* = 0.003)*, darkorange* (EV: *r* = 0.23, *p* = 0.4; cell: *r* =  − 0.77, *p* = 9e-05)*, darkgrey* (EV: *r* =  − 0.37, *p* = 0.1; cell: *r* = 0.58, *p* = 0.01)*, yellow* (EV: *r* =  − 0.37, *p* = 0.1 cell: *r* = 0.82, *p* = 7e-06)*, greenyellow* (EV: *r* =  − 0.56, *p* = 0.02; cell: *r* = 0.55, *p* = 0.02)*,* and *lightsteelblue* (EV: *r* =  − 0.58, *p* = 0.01; cell: *r* = 0.2, *p* = 0.4)). These data indicate that for 31% of Consensus modules, the enrichment of transcripts in EVs compared to cells varied from one pregnancy to another.

### Sex-dependent enrichment of RNA transcripts in EVs and cells

**Fig. 7:**
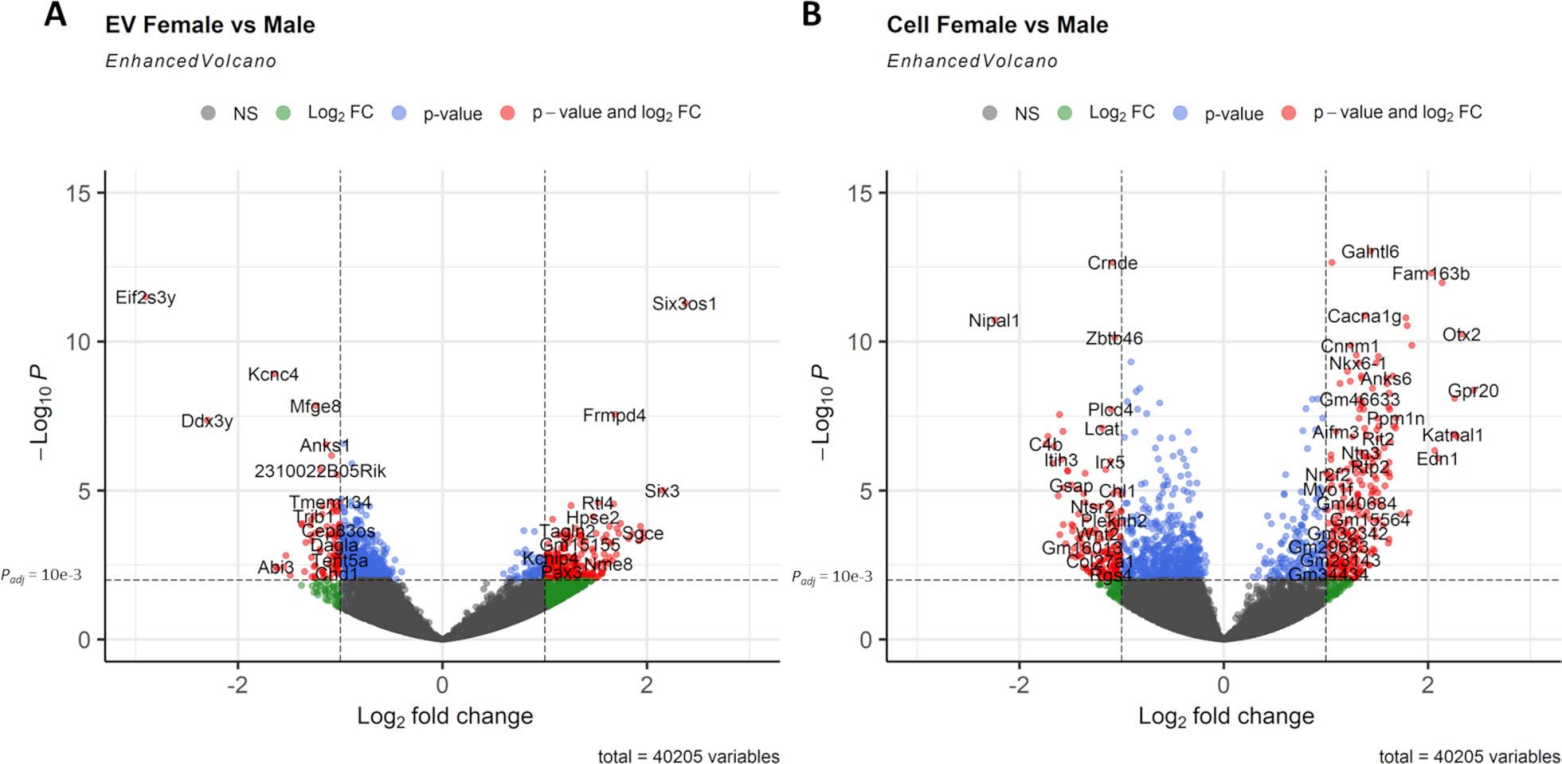
Differential Gene Expression of EV Samples and Cell Samples by Fetal Sex. **A**, **B** Volcano plots of log2 fold change and − log10 *p*-value of all genes differentially expressed in EV samples (**A**) or in cell samples (**B**) by female vs. male samples. Sample size for each comparison group, *n* = 9; dotted horizontal line is an adjusted p-value of 10e−03. Two dotted vertical lines are log2FC >|1|

**Fig. 8:**
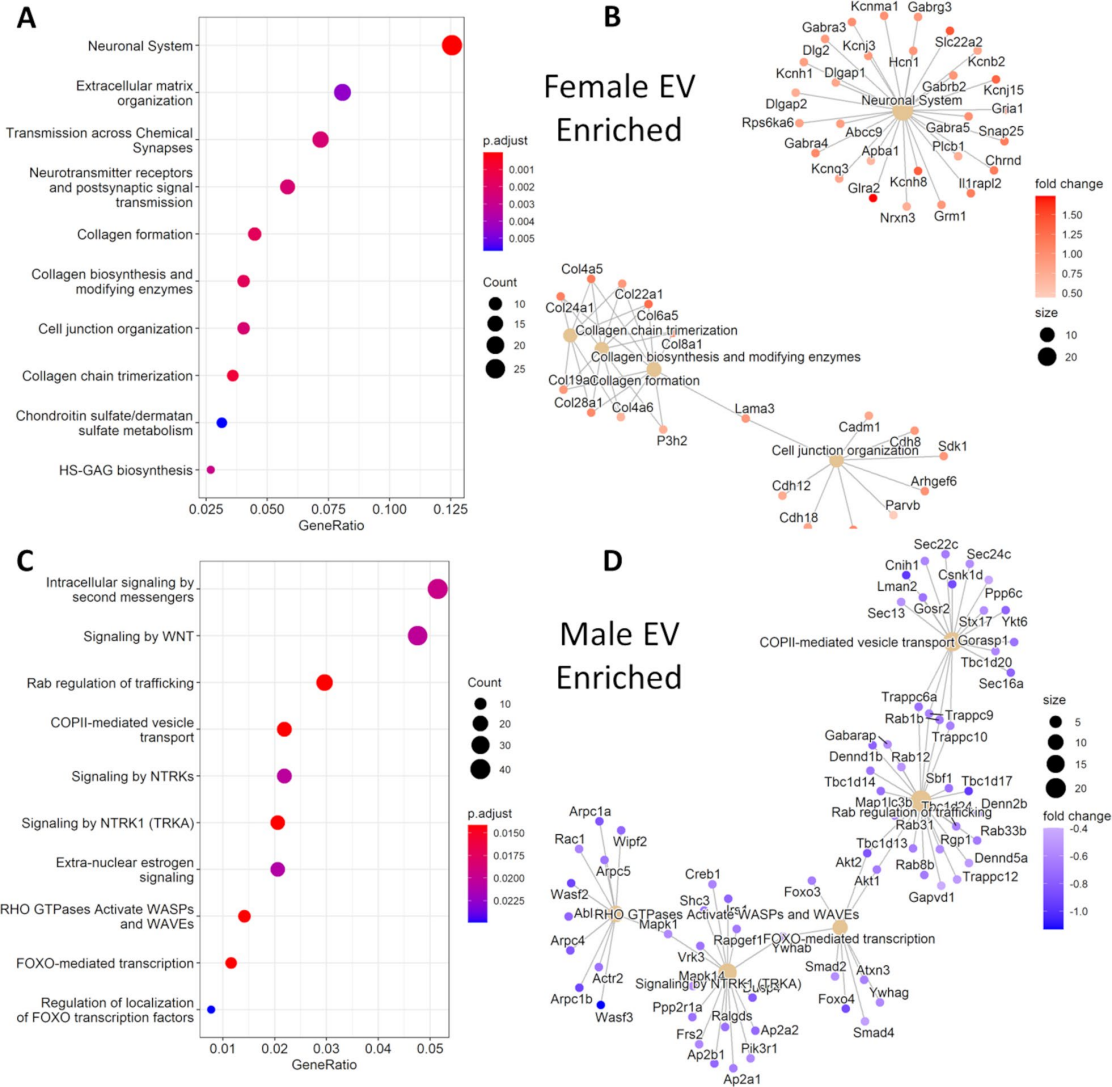
Pathway Overrepresentation Analysis of Enriched RNA Transcripts in EV Samples and Cell Samples by Sex. **A** Dot plot depicting pathway overrepresentation for significantly altered genes (adjusted *p* < 0.05) enriched in EV samples derived from their parental female NSCs (female EV) relative to EV samples derived from parental male NSCs (male EV) using *ReactomePA*. The plot presents overrepresented pathways, ordered by gene ratio, the proportion of differentially expressed genes/transcripts within an ontology term. The size of each dot denotes number of genes/transcripts in a pathway that were contained within this dataset, while the color of each dot encodes the Benjamini and Hochberg-adjusted *p*-value for significance of pathway overrepresentation. *n* = 9 female EV samples, 9 male EV samples. **B** Graphical representation of the relationship between enriched pathways and their associated genes/transcripts in female EVs. Significantly altered genes (adjusted *p* < 0.05) were selected for this analysis. Pathways that reached a Benjamini–Hochberg false discovery rate-adjusted p-value criterion of < 0.05 were selected. The size of each filled central circle represents the number of transcripts in a pathway that were overexpressed in female EVs. The color of each dot associated with that pathway denotes the fold change for that transcript. **C** pathway overrepresentation in male EV samples relative to female EV samples. **D** Graphical representation of the relationship between enriched pathways and their associated genes/transcripts in male EVs

As documented above, when all samples were combined for principal component analysis, the ‘*Location’* trait (EV vs. cell) well-defined the dominant source of variance between samples (principal component 1) and fetal sex well-defined the second largest source of variance (principal component 2) (Fig. 3A). For the 18 EV samples, DESeq2 analysis identified 2,859 significant sex-variant genes (DEGs; FDR‐corrected *p* < 0.05), including 1937 downregulated genes (67.75%) and 922 upregulated genes (32.25%) in female EV samples compared to male EV samples (Fig. 7A; Additional file 23: Table S17, Additional file 24: Table S18). For 18 cell samples, DESeq2 analysis identified 2,437 significant sex-variant genes (DEGs; FDR‐corrected *p* < 0.05), including 1,638 downregulated genes (67.21%) and 799 upregulated genes (32.79%) in female cell samples compared to male cell samples (Fig. 7B; Additional file 25: Table S19, Additional file 26: Table S20). To determine whether DEGs that were enriched by sex collectively served shared biological functions, we subjected the DEGs to pathway analysis (Fig. 8). For female EV-enriched DEGs, pathways associated with extracellular matrix organization and signal transmission were overrepresented (Fig. 8A, B; Additional file 27: Table S21). For male EV-enriched DEGs, pathways associated with intracellular signaling and vesicle transport were overrepresented (Fig. 8C, D; Additional file 28: Table S22). For female cell-enriched DEGs, a pathway associated with neuronal system was overrepresented (Additional file 6: Figure S6A, B; Additional file 29: Table S23). For male cell-enriched DEGs, pathways associated with RHO GTPase cycle and signaling by receptor tyrosine kinases were overrepresented (Additional file 6: Figure S6C, D; Additional file 30: Table S24). Overall, our data show a sex-dependent preferential transfer of distinctly different classes of genes, that are a part of largely non-overlapping biological pathways, from cell-of-origin NSCs to their secreted EVs.

### WGCNA for sex differences in transcriptomic profiles of EVs and cells

**Fig. 9:**
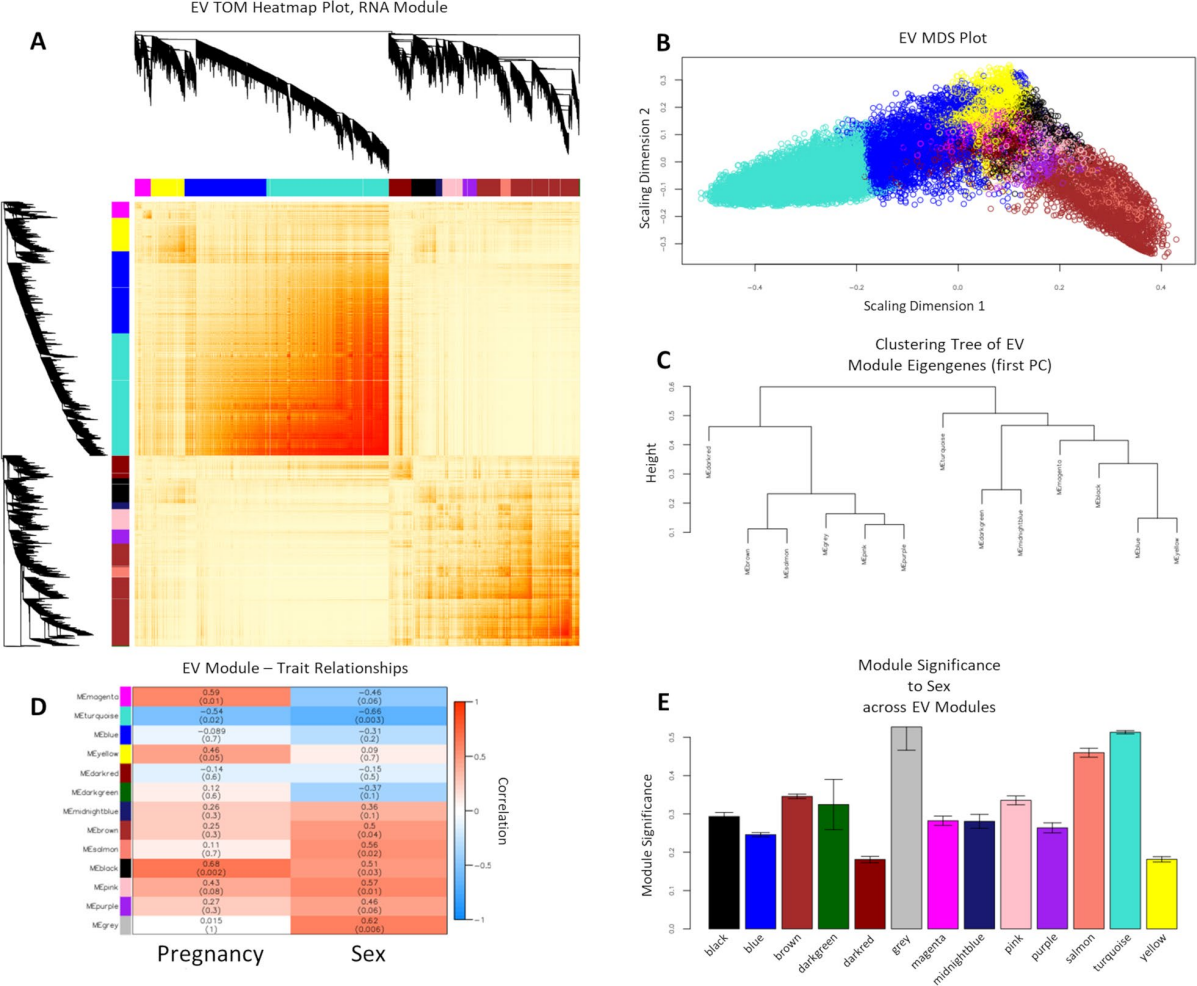
WCGNA-based Transcriptomic Profiling of EVs for Sex and Inter-Pregnancy Differences. WGCNA of RNA transcripts expressed in 16 EV samples, shows that EVs have distinct gene networks and correlations with the trait of ‘*Sex’* (female vs. male) contributing to the majority of the difference between clusters. The second trait, ‘*Pregnancy*’, was a smaller contributor to the overall composition of identified networks. **A** Topological overlap matrix (TOM) visualizing WCGNA in EVs. The *gray* module represents residual genes that could not be associated with networks. **B** Multidimensional Scaling (MDS) plot of genes in EVs show that the ‘*turquoise’* and ‘*brown’* module eigengenes are most separated from each other. **C** Module eigengene (ME) dendrogram as the dissimilarity measurement between MEs (1st principal component), showing the highest branching out of ME ‘*brown’* away from ME ‘*turquoise’*, also suggesting the greatest dissimilarity. **D** Heatmap of WGCNA module correlations with sample traits of ‘Pregnancy’ and ‘Sex’. The results of this analysis show that ‘*Sex’* (female vs. male) is the most important determinant of module identity, with the ‘*turquoise’* module identifying male, and the ‘*brown’*, ‘*salmon’*, ‘*black’*, and ‘*pink’* modules identifying female samples. The ‘*turquoise’* and ‘*black’* modules were also significantly associated with the trait of ‘*Pregnancy*’ and represent pregnancy-to-pregnancy variation in gene networks in EVs. **E** Bar plot of ‘*Sex’* (female vs. male) trait-based module significance (average gene significance in a module) across modules in EVs

For WGCNA in EVs, we used a topological overlap matrix (TOM) heatmap plot (Fig. 9A) and a multidimensional scaling (MDS) plot (Fig. 9B) to visualize the gene co-expression networks and similarity measurement of genes, and module eigengene (ME) dendrogram (Fig. 9C) as the dissimilarity measurement between modules/MEs. We observed highly correlated gene expression across *turquoise, blue, yellow*, and *magenta*, and highly correlated gene expression across *purple*, *pink*, *salmon*, *brown*, and *darkred* modules. We examined the correlation between EV-derived module MEs and sample traits of ‘Pregnancy’ and ‘*Sex’* (Fig. 9D). We found that there was a significant correlation between 6 of 12 MEs and sample trait ‘*Sex’*, with eigengene of *turquoise* (*r* =  − 0.66, *p* = 0.003) module having significant inverse correlation with ‘*Sex’,* i.e., higher in male relative to female samples, and eigengenes of *brown* (*r* = 0.5, *p* = 0.04), *salmon* (*r* = 0.56, *p* = 0.02), *black* (*r* = 0.51, *p* = 0.03), and *pink* (*r* = 0.57, *p* = 0.01) modules having significant positive correlations with ‘*Sex’*, i.e., higher in female relative to male samples. Then, we examined module significance (average absolute gene significance) across EV modules in relation to the sample trait ‘*Sex’* (Fig. 9E; Additional file 17: Table S11, Additional file 31: Table S25). As outlined in ‘Materials and methods’, to identify modules that are most relevant to the sample trait of interest, the module significance was calculated as the average gene significance (GS) of all genes in a module, while GS was defined as the absolute value of the correlation between the trait and a gene’s expression profile. For sample trait ‘*Sex’*, we identified the module significance of *turquoise* module (Module Significance: 0.51; Module Gene Count: 5430; Hub Gene: *Cenpa*), *brown* module (Module Significance: 0.34; Module Gene Count: 4069; Hub Gene: *Col24a1*), *salmon* module (Module Significance: 0.46; Module Gene Count: 452; Hub Gene: *Arhgap15*), *black* module (Module Significance: 0.29; Module Gene Count: 1081; Hub Gene: *Adam18*), and *pink* module (Module Significance: 0.34; Module Gene Count: 905; Hub Gene: *Kcnb2*) (Fig. 9E; Additional file 17: Table S11).

Next, we performed gene ontology enrichment analysis on the identified modules (Additional file 20: Table S14). We examined the gene ontology of modules with high module significance and high correlation to sample trait ‘*Sex’*, whose hub gene is significantly differentially expressed in female EV samples compared to male EV samples from the DESeq2 analysis of the 18 EV samples (Additional file 20: Table S17). The EV *turquoise* module had the highest module significance (0.51) (Additional file 20: Table S11) and the highest correlation (*r* =  − 0.66) (Fig. 9D) to sample trait ‘*Sex’*, with its hub gene *Cenpa,* Centromere Protein A, being one of the significant DEGs that is downregulated (log_2_(fold change) =  − 0.57; adjusted *p* = 0.04), or enriched in male EV samples compared to female EV samples (Additional file 24: Table S18). Hub gene *Cenpa,* Centromere Protein A, encodes a centromere protein containing a histone H3 related histone fold domain required for targeting to the centromere (Gene/Entrez ID 1058). Gene ontology of the *turquoise* module revealed that genes within this network are most associated with pathways involved in membrane-bounded organelle organization and cell-substrate adhesion (Additional file 20: Table S14). The EV *salmon* module had the highest module significance (0.45) (Additional file 17: Table S11) for a module with a positive correlation (*r* = 0.56) (Fig. 9D) to sample trait ‘*Sex’*, with its hub gene *Arhgap15,* Rho GTPase Activating Protein 15, being one of the significant DEGs that was upregulated (log_2_(fold change) = 1.35; adjusted *p* = 0.0002), meaning significantly enriched in female EV samples compared to male EV samples, from the DESeq2 analysis of 18 EV samples (Additional file 24: Table S18). Hub gene *Arhgap15,* encodes for the ARHGAP15 protein that can activate RHO GTPases to regulate diverse biological processes (Gene/Entrez ID 55,843). Gene ontology of the *salmon* module revealed that genes within this network are most associated with pathways involved in D5 dopamine receptor binding, and ectoderm formation (Additional file 24: Table S14).

**Fig. 10:**
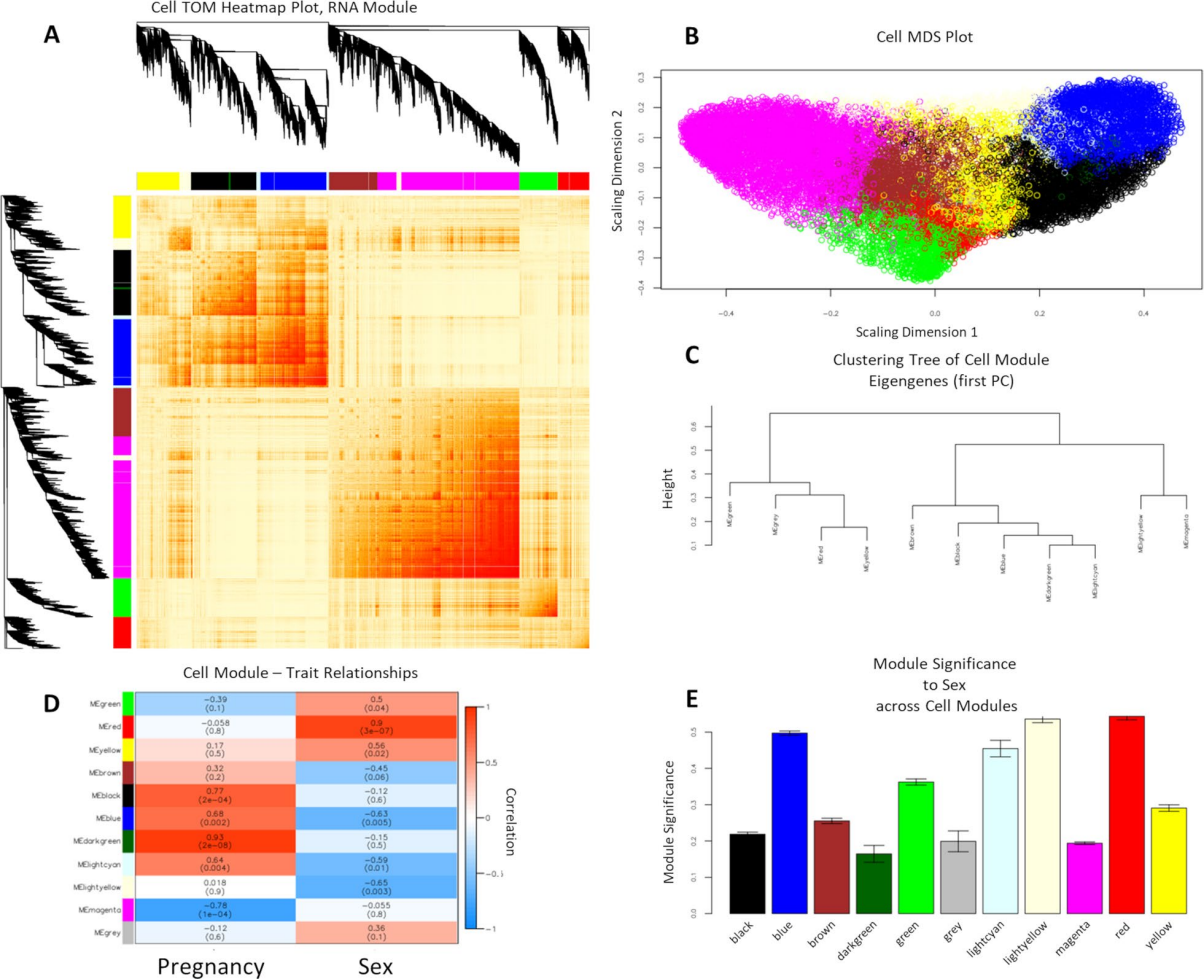
WCGNA-based Transcriptomic Profiling of Parent NSCs for Sex and Inter-Pregnancy Differences. WGCNA of RNA transcripts expressed in 16 cell samples, shows that parent NSCs have distinct gene networks and correlations with the trait of ‘*Sex’* (female vs. male) and ‘*Pregnancy*’ (cells derived from three separate pregnancies) contributing to the majority of the difference between clusters. **A** Topological overlap matrix (TOM) plot for visualizing the weighted gene co-expression network in NSCs. **B** Multidimensional Scaling (MDS) plot of genes in NSCs. **C** Module eigengene (ME) dendrogram as the dissimilarity measurement between MEs (1^st^ principal component), showing highest branching out of ME ‘*magenta*’ away from ME ‘*blue*’. **D** Heatmap of WGCNA module correlations with sample traits of ‘*Pregnancy*’ and ‘*Sex*’. The results of this analysis show that ‘*Sex’* (female vs. male) is the most important determinant of module identity, with the ‘*blue’*, ‘*lightcyan’*, and ‘*lightyellow’* module identifying male, and the ‘*green*’, ‘*red*’, and ‘*yellow*’ modules identifying female NSCs. **E** Bar plot of ‘*Sex’* (female vs. male) trait-based module significance (average gene significance in a module) across modules in NSCs

For WGCNA in cells, as before, we used TOM (Fig. 10A) and MDS (Fig. 10B) to visualize the gene co-expression networks, and the similarity measurement of genes and ME dendrogram (Fig. 10C) as the dissimilarity measurement between modules/MEs. We observed highly correlated gene expression between *black, blue,* and *yellow* modules, between the *brown* and *magenta* modules, and between *green* and *red* modules. We examined the correlation between cell-derived module MEs and sample traits of ‘Pregnancy’ and ‘*Sex’* (Fig. 10D). We observed that there was a significant correlation between 6 of 10 MEs and sample trait ‘*Sex’*, with eigengene of *blue* (*r* =  − 0.63, *p* = 0.005) *lightcyan* (*r* =  − 0.59, *p* = 0.01), and *lightyellow* (*r* =  − 0.65, *p* = 0.003) module having significant inverse correlation with ‘*Sex’*, i.e., higher in male relative to female cells, and eigengenes of *green* (*r* = 0.5, *p* = 0.04), *red* (*r* = 0.9, *p* = 3X10^−07^), and *yellow* (*r* = 0.56, *p* = 0.02) modules having significant positive correlations with ‘*Sex’*, i.e., higher in female relative to male cells. We then examined module significance across cell modules in relation to the sample trait ‘*Sex’* (Fig. 10E; Additional file 19: Table S13, Additional file 32: Table S26). For sample trait ‘*Sex’*, we identified the module significance of the *blue* (Module Significance: 0.5; Module Gene Count: 2889; Hub Gene: *Fbxo34*), *lightcyan* (Module Significance: 0.45; Module Gene Count: 249; Hub Gene: *Sgcb*), *lightyellow* (Module Significance: 0.54; Module Gene Count: 717; Hub Gene: *Actn4*), *green* (Module Significance: 0.36; Module Gene Count: 1675; Hub Gene: *n-TTagt6*), *red* (Module Significance: 0.54; Module Gene Count: 1383; Hub Gene: *Lhx4*), and *yellow* (Module Significance: 0.29; Module Gene Count: 1854; Hub Gene: *Parp11*) modules (Fig. 10E; Additional file 19: Table S13). Next, we performed gene ontology enrichment analysis on the identified modules (Additional file 21: Table S15). We examined the gene ontology of modules with high module significance and high correlation to sample trait ‘*Sex’*, whose hub gene is significantly differentially expressed in female cell samples compared to male cell samples from the DESeq2 analysis of 18 cell samples (Additional file 25: Table S19).

For the WGCNA of the cell data set, two modules had the highest module significance (0.54): *red* and *lightyellow* (Additional file 19: Table S13). The *red* module had the highest correlation (*r* = 0.9) (Fig. 10D) to sample trait ‘*Sex’*, with its hub gene *Lhx4,* LIM Homeobox 4, being one of the significant DEGs that is upregulated (log_2_(fold change) = 0.91; adjusted *p* = 4.43 × 10^–05^), or that this gene was enriched in female cell samples compared to male cell samples (Additional file 26: Table S20). *Lhx4* encodes a member of the LIM domain family of transcription factors that control of development and differentiation (Gene/Entrez ID 89,884). Gene ontology of the *red* module revealed that genes within this network are most associated with pathways involved in regulation of dense core granule transport and lysine methylation (Additional file 21: Table S15). *Lightyellow* had the strongest negative correlation (*r* =  − 0.65) (Fig. 10D) to sample trait ‘*Sex’*, with its hub gene *Actn4,* Actinin Alpha 4, significantly downregulated (log_2_(fold change) =  − 0.56; adjusted *p* = 0.03), or it was enriched in male cell samples compared to female cell samples, from the DESeq2 analysis of 18 cell samples (Additional file 25: Table S19). Hub gene *Actn4,* Actinin Alpha 4, encodes an actin-binding cytoskeletal protein associated with microfilament bundles and adherens-type junctions (Gene/Entrez ID 81). Gene ontology of the *lightyellow* module revealed that genes within this network are most associated with pathways for morphogenesis and regulation of dendrite development (Additional file 21: Table S15). Overall, our WGCNA and DESeq2 analyses of both EV and cell samples show that there are sex-dependent differences in the expression of individual genes as well as gene networks. Moreover, EVs contained a distinct and non-overlapping cluster of sex differences compared to their cell-of-origin NSCs.

### Ethanol exposure alters RNA expression level in EVs and in cells

**Fig. 11:**
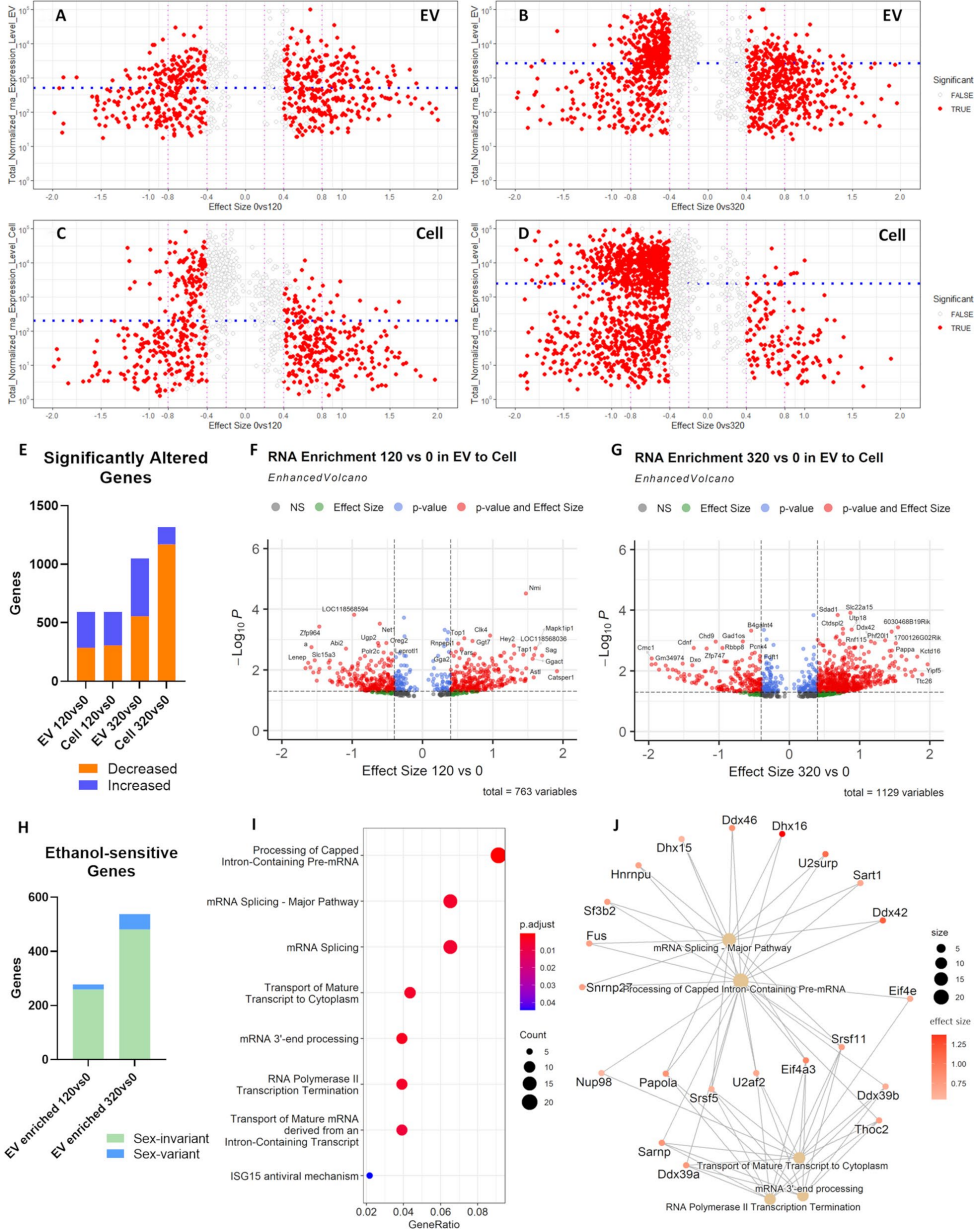
Effect of exposing NSCs to a Dose Range of Ethanol on the Transcriptome of Secreted EVs and Cells. **A**–**D** Scatter plots of effect size (Hedges’ g with nonzero-containing 95% Confidence Interval) vs. normalized RNA expression level, document that statistical significance (*p* < 0.05, red symbols) is independent of RNA expression (‘Y’ axis), but generally associated with decreased effect size. Relationship between effect size and statistical significance in EVs from 120 mg/dL-treated NSCs (**A**) or 320 mg/dL-treated NSCs (**B**) relative to control (0 mg/dL) NSCs. These analyses show that more differentially regulated EV transcripts from 320 mg/dL-treated NSCs also exhibited a negative effect size, indicative of downregulation in EVs. Relationship between effect size and statistical significance in parental NSCs treated with 120 mg/dL (**C**) or 320 mg/dL (**D**) relative to control (0 mg/dL) NSCs. Similar to EVs, these analyses show that more differentially regulated parental NSC transcripts also exhibited a negative effect size, indicative of downregulation. Horizontal green-dotted line represents the average transcript expression level of all transcripts, while horizontal blue-dotted line represents the average expression level of only transcripts for which the effect size had a non-zero containing 95% confidence estimate in EV (**A**, **B**) or parental cell (**C**, **D**) samples. Vertical purple-dotted lines denote effect size values of − 0.8, − 0.4, − 0.2, + 0.2, + 0.4, + 0.8, respectively. Positive effect size signifies increased expression of a transcript, and negative effect size signifies decreased expression of a transcript, in ethanol treatment group vs. control group. Each data point represents a transcript, with red color for significant p-value; n = 6 samples per group; paired *t*-test, *p* < 0.05. **E** A bar graph of the number of unique genes whose expression was significantly increased or decreased by ethanol. **F**, **G** Volcano plots of the relationship between effect size due to treatment and p-value. Each data point represents transcript enrichment in EVs relative to parental cells (EV/Cell) between moderate ethanol-treated (120 mg/dL, **F**) or heavy ethanol-treated (320 mg/dL, **G**) groups compared to the control group (0 mg/dL). These data show that transcripts that were significantly enriched in EVs due to heavy ethanol exposure were also depleted in parental NSCs. Blue triangles above the horizontal dotted line represent transcripts for which the effect size had a non-zero containing 95% confidence estimate that were also significantly affected by ethanol exposure by paired t-test.; n = 6 samples per group; paired *t*-test, *p* < 0.05. **H** A bar graph of the number of unique genes that are significant DEGs by sex (sex-variant) or not (sex-invariant) in EVs whose expression were significantly increased in EVs relative to cells by ethanol. **I** Pathway overrepresentation plots for transcripts that met the criteria for an effect size, >  + 0.4 for heavy ethanol exposure (320 mg/dL), a non-zero containing 95% confidence estimate and a significance of *p* < 0.05 by paired t-test. The size of each data point represents the number of genes/transcripts in a pathway that were within this subset of transcripts while color of data point denotes the FDR-corrected p-value for pathway overrepresentation. **J** Plot of key enriched pathways and constituent transcripts involved in these pathways, for heavy ethanol exposure. The size of each pathway element denotes the number of transcripts in a pathway that were within that subset of pathway transcripts, while the color of each element represents the effect size for that pathway component

While we found evidence for genetic sex as a large driver of EV transcriptomic differences, ethanol exposure also affected EV and cell transcriptomes. NSCs exposed to moderate (120 mg/dL) and heavy (320 mg/dL) levels of ethanol exposure exhibited significant and dose-related alterations in RNA transcripts, and moreover, their secreted EVs also exhibited ethanol-induced changes to their RNA transcriptome. For EVs, moderate and heavy ethanol exposure altered the expression of 591 and 1050 genes, respectively (using the combined criteria of paired *t*-test, *p* < 0.05; Hedges’ effect-size ‘g’ <  − 0.4 or ‘g’ >  + 0.4 with a non-zero-containing 95% confidence interval) (Fig. 11A, B; Additional file 33: Table S27, Additional file 34: Table 28). For the genes in EVs whose expression was significantly altered, 305 genes (~ 52%) following moderate ethanol exposure and 494 genes (~ 47%) following heavy ethanol exposure, were increased relative to controls. In contrast, for cells, moderate and heavy ethanol exposure altered 590 and 1315 genes, respectively, and the majority of them, 306 genes (~ 52%) in the moderate exposure group and 1171 genes (~ 89%) in the heavy exposure group, had lower expression than control-exposed NSCs (Fig. 11C, D; Additional file 35: Table S29, Additional file 36: Table 30). These analyses also indicate that while the majority (~ 89%) of differentially regulated cell-of-origin NSC transcripts from the heavy ethanol exposure group exhibited a negative effect size, indicative of downregulation, only ~ 53% of the differentially regulated EV transcripts from cell-of-origin NSCs treated with the moderate ethanol dose exhibited a negative effect size. This outcome raises the possibility that heavy ethanol exposure may increase the transfer of specific genes into EVs at the expense of cells, since the expression level of some gene transcripts was increased in EVs, while being decreased in cell-of-origin NSCs (Fig. 11E).

To test this hypothesis, we next assessed RNA enrichment in EVs by treatment groups (Fig. 11F, G), by calculating the EV-to-cell gene expression ratio (EV:cell) for each treatment group, followed by a paired comparison of the EV:cell ratio between each treatment group and the control group. For moderate ethanol exposure, the EV:cell ratio was significantly altered for 519 genes (using the combined criteria of paired *t*-test, *p* < 0.05; Hedges’ g < -0.4 or g >  + 0.4 with a non-zero containing 95% confidence interval), with the ratio significantly increased for ~ 54% (278) of the differentially altered genes relative to controls (Fig. 11F; Additional file 37: Table S31). In contrast, for heavy ethanol exposure, the EV:cell ratio was significantly altered for 734 genes, with 538 (~ 73%) significantly increased relative to control (Fig. 11G; Additional file 38: Table S32). Therefore, the heavy dose of ethanol resulted in preferential loading of specific ethanol-sensitive RNA transcripts into EVs at the expense of their intracellular levels in NSCs. Moreover, since this study was not powered to assess the interaction effect of ethanol and sex, we examined the overlap between ethanol-sensitive RNA transcripts and sex-specific DEGs and found only 6.8% (19 out of 278) of the genes that were significantly enriched in EVs following moderate ethanol exposure, were also significant DEGs by sex in EVs (Additional file 39: Table S33). In the case of the heavy ethanol exposure condition, we observed a marginal increase, 10.6% (57 out of 538) in genes that were significantly enriched in EVs were also significant DEGs by sex in EVs (Additional file 40: Table S34). Overall, a statistically significant majority of ethanol-sensitive RNAs that were enriched in EVs and depleted in cells were sex-invariant (*X*^2^_(3)_ = 10.05, *p* = 0.018, Fig. 11H).

To determine whether ethanol-sensitive RNA transcripts whose relative abundances were enriched in EVs collectively served shared biological functions, we subjected the EV-enriched ethanol DEGs to Gene Set Enrichment Analysis (GSEA). This analysis of significantly altered genes (FDR adjusted *p* < 0.05) identified significant biological pathways for heavy ethanol exposure, but not for the lower dose of ethanol. For heavy ethanol exposure, pathways associated with mRNA splicing/processing and transport of mature mRNA to cytoplasm were significantly overrepresented (Fig. 11I; Additional file 41: Table S35). Additional network analyses (Fig. 11J) documented the contribution of individual genes to core overrepresented pathways. Overall, our data show that ethanol exposure results in a dose-dependent preferential transfer of RNA transcripts, overrepresented for distinct biological pathways, from cell-of-origin NSCs to their secreted EVs.

### Confirmation of NSC expression of ethanol-sensitive EV RNA transcripts in vivo using scRNA-seq analysis of fetal ventricular zone cells

**Fig. 12:**
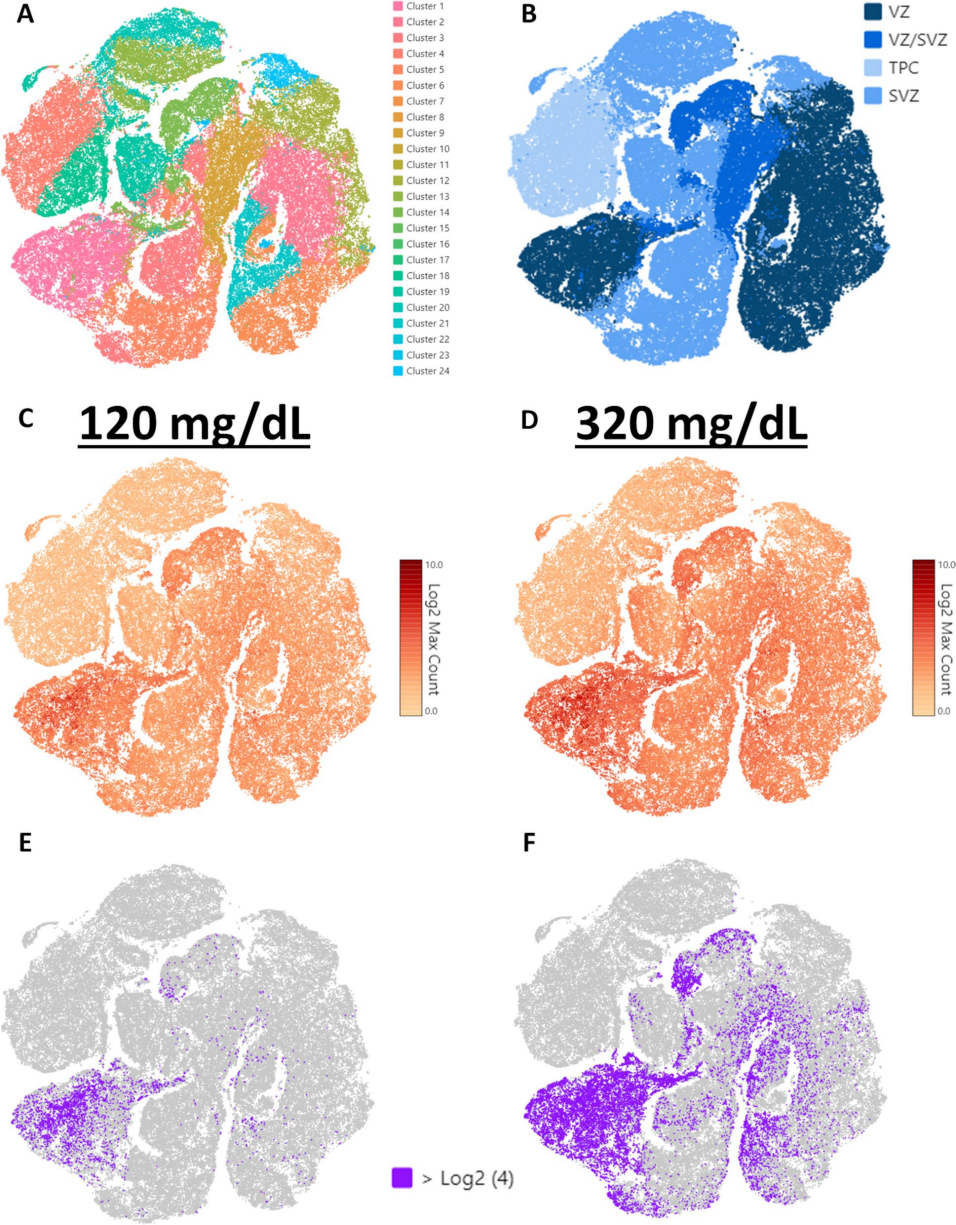
scRNAseq of GD 14.5 Murine Developing Cortex Shows that Ethanol-sensitive EV-Enriched RNA Transcripts are Abundant in Ventricular Zone Cell Lineages. **A** tSNE (t-distributed Stochastic Neighbor Embedding) plot of clusters identified as part of VZ, SVZ, or TPC lineages. Data extracted from NCBI/GEO (GSE158747). **B** tSNE plot classifying VZ, SVZ, TPC lineages as previously published [13]. Composite expression of mRNA transcripts which were significantly enriched in EVs obtained from 120 mg/dL (**C**) and 320 mg/dL (**D**)-treated NSCs; criterion cutoff of Hedges’ ‘g’ >  + 0.4. **E**, **F** The application of a threshold cutoff of > log_2_4 for composite transcript expression shows that in vivo, neural progenitor cells of the VZ are the principal contributors of RNA transcripts that are significantly enriched in EVs following exposure of parental NSCs to 120 mg/dL (**E**) or 320 mg/dL (**F**) of ethanol, with more VZ cell clusters expressing RNA transcripts that were enriched in EVs following heavy compared to moderate ethanol exposure

To confirm the expression of ethanol-sensitive EV RNA transcripts in NSCs in vivo, we examined their expression in our previously published scRNA-seq dataset (GSE158747) of GD14.5 fetal mouse cerebral cortical cells [13]. We focused on stem cell clusters (VZ, VZ/SVZ transition, SVZ, TPC) that had been previously reclustered [33] and were expected populations modeled with our in vitro neurosphere cultures (Fig. 12A, B). Pseudo-time analysis previously showed significant sex differences in maturation trajectories in VZ and SVZ subclusters, though each subcluster was present in both male and female fetal cortex [13]. However, for the purpose of defining the identity of these lineages, and mapping the cell-of-origin identity of ethanol-sensitive DEGs in EVs, data were collapsed across sex. We found that the ethanol-upregulated EV-enriched RNAs, with Hedges’ *g* > 0.4 effect size for moderate (120 mg/dL) or heavy (320 mg/dL) ethanol exposures, were not uniformly, nor globally, expressed throughout the fetal neurogenic niche (Fig. 12C, D), but were most abundant in the fetal mouse cortical VZ clusters (Fig. 12E, F). These data show that, among the different cell-type subpopulations in VZ, VZ/SVZ, SVZ, and TPC clusters, neural stem cells in the fetal VZ are the major cells-of-origin for RNA transcripts that are transferred from cells to EVs following ethanol exposure. Moreover, more cells within these clusters expressed EV-enriched genes that were elevated in response to the heavy ethanol dose.

## Discussion

EVs are a novel class of endocrine mediators with the potential to transfer macromolecules and information between cells and tissues. Our studies have focused on assessing whether EVs are also positioned to transmit the adverse effects of a perturbagen like ethanol from one cell to its neighbors as a means to reprogram early brain development. We recently showed that NSCs, obtained from the murine fetal dorsal telencephalon at the start of the neurogenic period and maintained ex vivo as neurosphere cultures, are remarkably active, releasing an estimated 10^3^ EVs/cell/day [32]. Moreover, EVs are rapidly and ubiquitously endocytosed by recipient NSCs in culture, where they influence important biological outcomes like the cell cycle, cellular respiration, and cell maturation [32].

Our previous study on mass-spectrometric mapping of the EV proteome [32] and the current RNAseq analysis of the EV transcriptome both show that the EV constitutes a distinct and unique compartment, and not merely a passive reflection of the contents of cell-of-origin NSCs. Both suggest the presence of active packaging mechanisms that selectively sort RNAs and proteins into EVs. Here we identified > 21,500 significant DEGs related to the trait of ‘*Location*’, i.e., differentially expressed in EVs compared to cell-of-origin NSCs, with 51.61% enriched in EVs compared to cell-of-origin NSCs. Pathway enrichment analysis of EV-enriched DEGs showed overrepresentation of pathways associated with intercellular transport whereas pathways associated with translation and cell cycle were enriched in cell-of-origin NSCs. WGCNA identified 12 unique gene network modules and their hub genes, the majority of which were significantly correlated with sample trait ‘*Location’*. Among these modules, 8 were enriched in EVs relative to cell-of-origin NSCs, including, for example, the ‘*lightcyan’* module which was enriched with genes for chaperone binding and protein sequestering activity. The hub gene *Ush2a*, whose disruption is associated with both autism spectrum disorders [76] and congenital sensorineural impairment [77], has itself been found to facilitate vesicle trafficking [78], consistent with the pathway identity for its EV-enriched module.

To further explore the differences between the transcriptomes of EVs and their cell-of-origin NSCs, we performed additional WGCNA to identify consensus modules, i.e., genes densely connected in both EV and cell samples, and compared those consensus modules to EV WGCNA-identified and cell WGCNA-identified modules. Most EV WGCNA-identified and cell WGCNA-identified modules had multiple consensus counterparts, indicating that gene network structure in these modules were similar in EVs and NSCs. However, the EV WGCNA-identified module *darkgreen* (different from the cell *darkgreen* module) had no consensus counterpart and was enriched for pathways for RNA polymerase I transcription activity involved in cell proliferation. Another EV WGCNA-identified module *midnightblue* module had only one consensus counterpart and was enriched for pathways in meiotic division and cell differentiation. These data collectively indicate that EVs are specifically enriched in transcripts that support NSC proliferation and differentiation. A somewhat surprising finding was that the RNA content in EVs did exhibit some pregnancy-related variation, i.e., NSCs from one pregnancy to the next produced EVs with a somewhat different complement of RNAs. This outcome occurred despite NSCs from different pregnancies being maintained ex vivo, in an identical culture environment to reduce technical variabilities. While it is possible that differences in cell culture conditions may have contributed to some difference between pregnancies, it should be noted that cells obtained from each pregnancy were cultured in temporal contiguity. Moreover, the similarity of the RNA content of the cell-of-origin NSCs derived from different pregnancies argues against a contribution from technical variations in culture conditions. Rather, pregnancy-to-pregnancy variation in EVs suggests the possibility of an epigenetic imprint of each pregnancy on NSCs derived from that pregnancy. To our knowledge, pregnancy-to-pregnancy variation in EV content has not been documented before, particularly in inbred mouse strains. However, other within-strain variations, for example, in behavioral phenotypes like alcohol preference and linked gene expression differences have been previously observed [79] and are attributable to variation in environmental influences. Interpregnancy differences in EV content may be similarly driven by subtle differences in the environment from one pregnancy to the next.

In this study, we also identified robust sex differences in the transcriptome of EVs, especially compared to their cell-of-origin NSCs. This outcome was all the more surprising, because we did not previously see a similar effect of genetic sex on the protein content of secreted EVs [32]. Following DESeq2 analysis, we identified 2,859 sex-dependent DEGs in EV samples. A majority, ~ 68%, or 1,937 DEGs represented significantly higher gene expression in male compared to female EVs, while only 32% of DEGs represented transcripts that were significantly elevated in female EV samples. These data suggest that male EVs express a greater variety and abundance of RNAs than female EVs. Moreover, male-enriched DEGs in EVs were associated with intracellular signaling and vesicle transport, whereas female-enriched DEGs in EVs were associated with extracellular matrix organization and signal transmission. These outcomes suggest that male and female EVs may support different biological outcomes in the event that their RNA cargo is delivered to recipient cells and tissues. A second finding was that the sex-dependent DEGs in EVs were different from those in their cell-of-origin NSCs. For instance, we identified ~ 2,400 sex-dependent DEGs in NSCs that were preferentially increased in male NSCs, ~ 67%, compared to female NSCs, which is a similar percentage as sex-dependent DEGs that are enriched in male EV samples compared to female EV samples. However, sex-dependent DEGs enriched in male NSCs formed distinctly different networks, with overrepresentation of RHO GTPase and receptor tyrosine kinase pathways for example, compared to sex-dependent DEGs enriched in male EVs. WGCNA also identified sex-dependent EV-specific gene network modules that were distinct from cell-specific modules, each with different hub genes and mostly non-overlapping biological pathways. In addition, we followed up the primary WGCNA analysis by assessing the relationship between WGCNA-derived EV-Cell consensus modules and sample trait ‘*Sex’*. In this analysis, we found that many consensus modules were significantly correlated with sample trait ‘*Sex’*. This indicates that the gene co-expression pattern in the associated consensus modules was dependent on fetal sex. Additionally, some consensus modules were dependent on both the traits of ‘*Sex*’ and ‘*Location’*. In these consensus modules, for example, networks of correlated transcripts were more highly expressed in female EVs compared to male EVs, but expressed at lower levels in female cell-of-origin NSCs compared to male NSCs. The biological implications of these findings need further investigation, but they suggest an active sex-dependent sequestration of networks of RNAs from NSCs to secreted EVs, and that EVs are not a passive mirror of the RNA content of their cell-of-origin NSCs.

Other groups have previously reported on sex differences in the transcriptome of the developing brain [80], and more recently, we also identified sex differences in maturation trajectories in fetal mouse brain using single cell transcriptome analysis [13, 80]. It is likely that intrinsic sex differences in the transcriptome of fetal neural cells may translate into sex differences in their secretome as well. However, only a few studies have focused on inherent sex differences in secreted EVs. For instance, synovial exosome from joints have been found to express sex-specific patterns of miRNAs predictive of osteoarthritis [81]. A more recent study showed that there were sex differences in sub-cellular compartment protein representation in EVs derived from adult mouse brain [82]. Finally, in a recent study [83] that is pertinent to the outcomes of the current study, Baratta and colleagues documented sex differences in RNA content of EVs derived from whole brain of adult control mice, or mice exposed to alcohol. It was interesting to note that ~ 12% of transcripts that exhibited sex-dependent expression in EVs in our study, also exhibited sex-dependent expression in the study by Baratta and colleagues. It is likely that both tissue source, i.e., purified NSCs vs. whole brain (which contains only a minor NSC fraction) and developmental stage, i.e., fetus vs. adult contribute substantially to sex differences in the transcriptome of neural EVs. However, collectively, these studies suggest that sex differences in the content of EVs are likely to be pervasive and ethanol exposure may differently affect sex-dependent RNA content of EVs that control sexually distinct biological processes in the brain, but need additional investigation. Importantly, there needs to be careful assessment of the relationship between sex differences in EVs to their cell-of-origin NSCs. Our data suggest that EVs differ from their cell-of-origin NSCs quite substantially, and therefore, exhibit a novel cluster of transcriptome-based sex differences compared to their cell-of-origin NSCs.

The initial goal of these studies was to understand the effects of ethanol on RNA transcript sequestration into EVs. Cell-of-origin NSCs maintained ex vivo as neurosphere cultures were exposed to control media or to one of two doses of ethanol, both within a range consumed in human populations, especially by persons with alcohol use disorders [55]. Within the cell-of-origin NSCs, we observed a dose-related decrease in transcript expression, i.e., an increase in the number of significantly down-regulated genes, following ethanol exposure, and at the highest dose, ~ 89% of all differentially regulated genes were downregulated compared to controls. However, the outcome for EVs was surprisingly quite different. Here we observed that depending on the dose, between ~ 47% and ~ 52% of significantly differentially regulated genes were enriched due to ethanol exposure compared to controls. This outcome runs counter to the possibility that ethanol toxicity simply results in decreased transcription. Rather, this outcome suggests that NSCs may respond to ethanol exposure by transferring RNA transcripts into EVs.

This hypothesis was at least partly borne out by our analyses. For instance, exposure of NSCs to the highest dose of ethanol, resulted in the production of EVs, where the EV-to-cell gene expression ratio was significantly altered for 734 genes, with about 73% of them being significantly increased relative to controls, i.e., overrepresented in EVs compared to cell-of-origin cells. Moreover, these RNA transcripts that exhibited preferential ethanol-stimulated loading into EVs compared to their cell-of-origin cells were overrepresented in specific pathways associated with mRNA splicing/processing and transport of mature mRNA to cytoplasm. Since this effect of EV-enrichment was not generalized to all RNA transcripts present in EVs, these data suggest the possibility of a novel and selective ethanol-dependent mechanism that controls RNA sorting into EVs. This hypothesis of selective sorting is supported by our finding that ~ 16% and ~ 21% of EV-enriched transcripts in the moderate and high ethanol exposure conditions, respectively, encoded nuclear-localized proteins as defined by the ‘Compartments’ sub-cellular localization database [84]. In contrast, very few transcripts encoding other sub-cellular organelle-localized proteins were enriched in EVs derived from ethanol-treated NSCs. Interestingly, a reanalysis of a previously published scRNAseq study of the developing fetal murine cerebral cortex [13] showed that ethanol-sensitive transcripts that were preferentially enriched in EVs relative to cell-of-origin cells following ethanol exposure were unevenly expressed in the neurogenic niche, heavily localized to VZ-type cells rather than to SVZ and TPC clusters (Fig. 12). This raises the possibility that EVs are positioned to transfer transcripts from one NSC to another or across stages of maturation.

Though EVs can mediate intercellular communication [32], it is not clear whether the preferential loading of RNA transcripts from NSCs to EVs represents an adaptive, transcript shedding response to a stressor, or a means for information transfer. For example, Kctd16 (Potassium Channel Tetramerization Domain Containing 16), a transcript that exhibited a shift from cells to EVs at both doses of ethanol, encodes an auxiliary subunit of the GABA_B_ metabotropic receptor [85]. Kctd16 belongs to a family of proteins, including KCtd12 that was enriched in EVs following exposure to the high dose of ethanol, that facilitates rapid GABA_B_ receptor desensitization [86]. The GABA_B_ receptor has been shown to promote both neuronal migration and differentiation during development [87]. Therefore, shedding essential transcripts for GABA_B_ desensitization may result in aberrant maturation of NSCs, as we have previously observed [15, 18, 20, 33]. An equally plausible alternate hypothesis is that this ethanol-dependent RNA transfer supports the emergence of new endocrine biology, where EVs are positioned to modify the transcriptome and proteome of recipient neural cells. As an example, NMI (N-Myc and STAT Interactor), a top candidate transcript that was enriched in EVs at the expense of cells following moderate ethanol exposure, encodes a protein that has been implicated in cell cycle inhibition [88] and is also a danger-associated molecular pattern (DAMP) signal [89], associated with inflammation and the innate immune response. The shedding of NMI and related transcripts may contribute to the observed increase in cell cycle in NSCs following ethanol exposure [20], but may also communicate inflammation signals via DAMP pathways that have previously been linked with prenatal alcohol exposure [90].

This study had a number of strengths, including sampling from multiple pregnancies and from sex-specified neural progenitor cells expanded ex vivo. To our knowledge, this is also the first study to compare the transcriptome of EVs to that of their cell-of-origin NSCs to uncover an apparent shift in the localization of transcripts from cell-of-origin cells to EVs under the stimulus of an environmental perturbagen, ethanol. This is one of very few studies to also specifically address the substantial contribution of biological sex to the contents of EVs and to also show that RNA contents of EVs exhibited some variance from one pregnancy to another. However, there were also weaknesses in this study. To generate sufficient quantities of EVs for RNAseq analysis, we chose an intermediate ex vivo model, where primary cells were indeed derived from microdissected fetal murine cortex, but then expanded under well-defined culture conditions to generate the required quantities of sample material. Though an in vivo, whole animal model would be preferred, it should be noted that comparisons of the ethanol-responsive transcriptome in the current study with transcript data from our previously published scRNAseq dataset obtained from the analysis of cells directly micro-dissected from fetal dorsal telencephalon [13] confirmed that ethanol-responsive RNA transcripts in EVs can be mapped to fetal ventricular zone NSCs (Fig. 12). Another important caveat is that native EVs secreted by NSCs appear to contain mainly ribosomal RNAs. The significance of this preference is unknown, but to more carefully assess the transcriptome of EVs, we depleted the ribosomal RNA content of both EVs and their cell-of-origin cells. It should be noted that ribosomal depletion has been suggested as a cost-efficient approach for RNAseq studies in cells and tissues [91], and is practiced widely [92], but this choice may add systematic bias to the outcomes reported in this study. Nevertheless, the main outcomes of this study, that the transcriptome of EVs differs significantly from their cell-of-origin NSCs, that biological sex contributes to variation in the RNA content of EVs, and that ethanol, an important teratogen, appears to result in a selective enrichment of transcripts serving specific pathways into EVs at the expense of their cell-of-origin NSCs, are important and support the need for further investigation.

## Perspectives and significance

EVs are a novel mechanism for the intercellular transfer of proteins and RNAs. We observed that the transcript profile of ribosomal RNA-depleted EVs was distinctly different from that of ribosomal RNA-depleted cell-of-origin NSCs. Moreover, different networks of genes contributed to sex differences in the transcriptome of NSCs compared to their secreted EVs, suggesting that sex-specific mechanisms may contribute to the sequestration of RNA transcripts into EVs. Exposing cell-of-origin NSCs to ethanol, an important developmental teratogen, resulted in a dose-related increase in RNA transcripts in EVs and a decrease in those transcripts in cell-of-origin NSCs. This suggests that ethanol exposure resulted in a re-sorting of RNA transcripts from cell-of-origin NSCs to secreted EVs. EV-enriched transcripts due to ethanol exposure were overrepresented in specific pathways associated with mRNA splicing, processing and transport, suggesting the selective shedding of encoded RNA processing machinery by cell-of-origin NSCs due to ethanol exposure. Understanding the role of EVs in response to ethanol in NSCs may contribute to our understanding of the etiology of neurodevelopmental disorders, including those due to prenatal alcohol exposure.

## Supplementary Information

**Additional file 1: Fig. S1.** RNA quality analysis and Aligned RNA Base Distribution without Ribosomal RNA Depletion. A) Agilent tape station analysis of RNA size distribution in cell-of-origin NSCs and secreted EVs. B) Preliminary RNAseq study of total RNA library prepared without ribosomal RNA depletion of 5 EV samples. In the absence of ribosome RNA depletion, the majority of reads map to ribosomal RNAs.

**Additional file 2: Fig. S2.** Principal Component Analysis of Cell and EV Samples Grouped by Pregnancy. Principal component analysis was performed on the 500 most variant RNA transcripts from 18 NSC parental cell samples (S2A) and their corresponding EV samples (S2B). This analysis shows that parental NSC samples, but not their secreted EVs, could be partly segregated within the 1st two principal components, by their pregnancy identity (i.e., which pregnant dam the fetal cells were derived from). This finding guided this study’s use of a repeated measures experimental design with pregnancy ID as a within-subjects factor for parametric statistical analyses.

**Additional file 3: Fig. S3.** Direct Fluorescent Labeling of NSCs with PKH26. Confocal photomicrograph of NSCs that were directly labeled with PKH26 as a positive control with a paired phase-contrast image of a single neurosphere; PKH26-labeled NSCs are shown in red.

**Additional file 4: Fig. S4.** Control for Specificity of Fluorescent Labeling Protocol for EVs. Confocal photomicrograph of a negative control, naïve NSCs administered culture medium spiked with PKH26 dye, but subjected to the identical labeling and filtration process as that used for labeling of isolated EVs. This study shows that residual dye is removed by the purification process for EV labeling, and that any fluorescence in recepient cells is due to uptake of labeled EVs (as shown in Fig. 2). NSC nuclei are counter-stained with DAPI (blue fluorescence).

**Additional file 5: Fig. S5.** RNA transcripts as contributing variables to PCA. Principal component analysis was performed on the 500 most variant RNA transcripts from 18 NSC parental cell samples and their corresponding 18 EV samples. This figure labels RNA transcripts (a full list in Additional file 8: Table S2) that are contributing variables to the principal component analysis, and is supplemental to Fig. 3A, which showed that the samples could be segregated with the 1^st^ principal component, by their sample type (whether the RNA transcripts are from EV or cell sample).

**Additional file 6: Fig. S6.** Pathway Overrepresentation Analysis of Enriched RNA Transcripts in Cell Samples by Sex. A,C) Dot plot depicting pathways related to significantly altered genes (adjusted p < 0.05) enriched in A) female cell samples relative to male cell samples, C) male cell samples relative to female cell samples, as revealed by *ReactomePA*. The plot presents overrepresented pathways, ordered by gene ratio, the proportion of differentially expressed genes/transcripts within an ontology term. The size of each dot denotes number of genes/transcripts in a pathway that were contained within this dataset, while the color of each dot encodes the Benjamini and Hochberg-adjusted p-value for significance of pathway overrepresentation. n = 9 female cell samples, 9 male cell samples. B,D) The figures graphically represent the relationship between enriched pathways and their associated genes/transcripts. Significantly altered genes (adjusted p < 0.05) were selected for this analysis. Pathways that reached a Benjamini–Hochberg false discovery rate-adjusted p-value criterion of < 0.05 were selected. The size of each filled central circle represents the number of transcripts in a pathway that were overexpressed in female cells (B), or male cells (D). The color of each dot associated with that pathway denotes the fold change for that transcript in female relative to male.

**Additional file 7: Table S1.** RNA-seq Gene Counts of 36 Samples DESeq2-normalized HTSeq gene counts of all 18 EV and 18 cell samples.

**Additional file 8: Table S2.** Principal Component Analysis of 36 Samples Principal component analysis of the 500 most variant RNA transcripts from 36 samples.

**Additional file 9: Table S3.** RNA Transcript Enrichment by EV vs. Cell, DESeq2 Analysis of 36 Samples DESeq2 analysis on the distribution of RNA transcript enrichment by sample trait ‘*Location’* (EV vs. cell) on 36 samples to identify significant differentially expressed genes (DEGs; adjusted p < 0.05; Benjamini and Hochberg method).

**Additional file 10: Table S4.** Reactome Pathway Analysis of RNA Transcripts Enriched in EVs DESeq2 analysis identified significant differentially expressed genes (DEGs) that were enriched in EVs relative to cells (adjusted p < 0.05; Benjamini and Hochberg method). Pathway overrepresentation analysis was conducted using the Reactome pathway database (reactome.org).

**Additional file 11: Table S5.** Reactome Pathway Analysis of RNA Transcripts Enriched in Cells DESeq2 analysis identified significant differentially expressed genes (DEGs) that were enriched in cells relative to EVs (adjusted p < 0.05; Benjamini and Hochberg method). Pathway overrepresentation analysis was conducted using the Reactome pathway database (reactome.org).

**Additional file 12: Table S6.** WGCNA Gene Information of 36 Samples A table of each gene’s Gene ID, Entrez ID, and module color.

**Additional file 13: Table S7.** WGCNA Modules and Hub Genes of 36 Samples A table of each module’s color, gene count, module significance to trait ‘*Location’*, hub gene, and hub gene’s entrez ID from WGCNA, and hub gene’s log2(Fold Change) of EV vs. cell and adjusted p-value from DESeq2 analysis.

**Additional file 14: Table S8.** WGCNA Gene Information of 36 Samples in Relation to Sample Trait Location A table of each gene’s module color, gene significance to trait ‘*Location’*, and module membership.

**Additional file 15: Table S9.** WGCNA Gene Ontology Enrichment Table of 36 Samples Enrichment analysis of genes in all modules to study biological mechanisms.

**Additional file 16: Table S10.** WGCNA Gene Information of EV Samples A table of each gene’s Gene ID, Entrez ID, and module color from 18 EV samples.

**Additional file 17: Table S11.** WGCNA Modules and Hub Genes of EV Samples A table of each module’s color, gene count, module significance to trait ‘*Sex*’, hub gene, and hub gene’s entrez ID from WGCNA of EV samples, and hub gene’s log2(Fold Change) of female vs. male and adjusted p-value from DESeq2 analysis of EV samples.

**Additional file 18: Table S12.** WGCNA Gene Information of Cell Samples A table of each gene’s Gene ID, Entrez ID, and module color from 18 cell samples.

**Additional file 19: Table S13.** WGCNA Modules and Hub Genes of Cell Samples A table of each module’s color, gene count, module significance to trait ‘*Sex*’, hub gene, and hub gene’s entrez ID from WGCNA of cell samples, and hub gene’s log2(Fold Change) of female vs. male and adjusted *p*-value from DESeq2 analysis of cell samples.

**Additional file 20: Table S14.** WGCNA Gene Ontology Enrichment Table of EV Samples Enrichment analysis of genes in all modules from EV samples to study biological mechanisms.

**Additional file 21: Table S15.** WGCNA Gene Ontology Enrichment Table of Cell Samples Enrichment analysis of genes in all modules from cell samples to study biological mechanisms.

**Additional file 22: Table S16.** WGCNA Gene Information of Consensus Module Analysis A table of each gene’s consensus module color and gene significance to sample traits ‘*Sex*’, ‘Pregnancy’, and ‘*Alcohol*’ from consensus analysis of combined network.

**Additional file 23: Table S17.** RNA Transcript Enrichment by Sex, DESeq2 Analysis of EV Samples DESeq2 analysis on the distribution of RNA transcript enrichment by sample trait ‘*Sex*’ (female vs. male) on 18 EV samples to identify significant differentially expressed genes (DEGs; adjusted p < 0.05; Benjamini and Hochberg method).

**Additional file 24: Table S18.** Significant DEGs by Sex in EV Samples Significant DEGs by sample trait ‘*Sex*’ (female vs. male) on 18 EV samples identified by DESeq2 analysis (adjusted p < 0.05; Benjamini and Hochberg method).

**Additional file 25: Table S19.** RNA Transcript Enrichment by Sex, DESeq2 Analysis of Cell Samples DESeq2 analysis on the distribution of RNA transcript enrichment by sample trait ‘*Sex*’ (female vs. male) on 18 cell samples to identify significant differentially expressed genes (DEGs; adjusted p < 0.05; Benjamini and Hochberg method).

**Additional file 26: Table S20.** Significant DEGs by Sex in Cell Samples Significant DEGs by sample trait ‘*Sex*’ (female vs. male) on 18 cell samples identified by DESeq2 analysis (adjusted p < 0.05; Benjamini and Hochberg method).

**Additional file 27: Table S21.** Reactome Pathway Analysis of RNA Transcripts Enriched in Female EVs DESeq2 analysis identified significant differentially expressed genes (DEGs) that were enriched in female EVs relative to male EVs (adjusted p < 0.05; Benjamini and Hochberg method). Pathway overrepresentation analysis was conducted using the Reactome pathway database (reactome.org).

**Additional file 28: Table S22.** Reactome Pathway Analysis of RNA Transcripts Enriched in Male EVs DESeq2 analysis identified significant differentially expressed genes (DEGs) that were enriched in male EVs relative to female EVs (adjusted p < 0.05; Benjamini and Hochberg method). Pathway overrepresentation analysis was conducted using the Reactome pathway database (reactome.org).

**Additional file 29: Table S23.** Reactome Pathway Analysis of RNA Transcripts Enriched in Female Cell DESeq2 analysis identified significant differentially expressed genes (DEGs) that were enriched in female cell relative to male cell (adjusted p < 0.05; Benjamini and Hochberg method). Pathway overrepresentation analysis was conducted using the Reactome pathway database (reactome.org).

**Additional file 30: Table S24.** Reactome Pathway Analysis of RNA Transcripts Enriched in Male Cell DESeq2 analysis identified significant differentially expressed genes (DEGs) that were enriched in male cell relative to female cell (adjusted p < 0.05; Benjamini and Hochberg method). Pathway overrepresentation analysis was conducted using the Reactome pathway database (reactome.org).

**Additional file 31: Table S25.** WGCNA Gene Information of EV Samples in Relation to Sample Trait Sex A table of each gene’s module color, gene significance to trait ‘*Sex*’, and module membership for EV samples.

**Additional file 32: Table S26.** WGCNA Gene Information of Cell Samples in Relation to Sample Trait Sex A table of each gene’s module color, gene significance to trait ‘*Sex*’, and module membership for cell samples.

**Additional file 33: Table S27.** Effects of Moderate Ethanol Exposure on EV RNA Expression Table of moderate ethanol exposure-sensitive RNA transcripts in EV samples with gene name, p-value (paired *t*-test) and effect size (Hedges’ g) of moderate (120 mg/dL) and heavy (320 mg/dL) ethanol-treated groups, EVs’ mean values of control, moderate, and heavy ethanol-treated groups, and total RNA expression level in EV samples; paired *t*-test; Hedges’ g with 95% confidence estimate.

**Additional file 34: Table S28.** Effects of Heavy Ethanol Exposure on EV RNA Expression Table of heavy ethanol exposure-sensitive RNA transcripts in EV samples with gene name, p-value (paired *t*-test) and effect size (Hedges’ g) of moderate (120 mg/dL) and heavy (320 mg/dL) ethanol-treated groups, EVs’ mean values of control, moderate, and heavy ethanol-treated groups, and total RNA expression level in EV samples; paired *t*-test; Hedges’ g with 95% confidence estimate.

**Additional file 35: Table S29.** Effects of Moderate Ethanol Exposure on Cell RNA Expression Table of moderate ethanol exposure-sensitive RNA transcripts in cell samples with gene name, p-value (paired *t*-test) and effect size (Hedges’ g) of moderate (120 mg/dL) and heavy (320 mg/dL) ethanol-treated groups, cells’ mean values of control, moderate, and heavy ethanol-treated groups, and total RNA expression level in cell samples; paired *t*-test; Hedges’ g with 95% confidence estimate.

**Additional file 36: Table S30.** Effects of Heavy Ethanol Exposure on Cell RNA Expression Table of heavy ethanol exposure-sensitive RNA transcripts in cell samples with gene name, p-value (paired *t*-test) and effect size (Hedges’ g) of moderate (120 mg/dL) and heavy (320 mg/dL) ethanol-treated groups, cells’ mean values of control, moderate, and heavy ethanol-treated groups, and total RNA expression level in cell samples; paired *t*-test; Hedges’ g with 95% confidence estimate.

**Additional file 37: Table S31.** Effects of Moderate Ethanol Exposure on RNA Enrichment Table of RNA transcripts to measure ethanol effects on RNA enrichment in EVs by EV/Cell ratio between control group and moderate ethanol-treated (120 mg/dL) group; paired *t*-test, *p* < 0.05; Hedges’ g with 95% confidence estimate.

**Additional file 38: Table S32.** Effects of Heavy Ethanol Exposure on RNA Enrichment Table of RNA transcripts to measure ethanol effects on RNA enrichment in EVs by EV/Cell ratio between control group and heavy ethanol-treated (320 mg/dL) group; paired *t*-test; Hedges’ g with 95% confidence estimate.

**Additional file 39: Table S33.** Sex-variant Effects of Moderate Ethanol Exposure on RNA Enrichment Table of significant DEGs by sex in EVs that are also enriched in EVs by EV/Cell ratio between control group and moderate ethanol-treated (120 mg/dL) group; paired *t*-test; Hedges’ g with 95% confidence estimate.

**Additional file 40: Table S34.** Sex-variant Effects of Heavy Ethanol Exposure on RNA Enrichment Table of significant DEGs by sex in EVs that are also enriched in EVs by EV/Cell ratio between control group and heavy ethanol-treated (320 mg/dL) group; paired *t*-test; Hedges’ g with 95% confidence estimate.

**Additional file 41: Table S35.** Reactome Pathway Analysis of EV-Enriched RNA Transcripts Sensitive to Heavy Ethanol Exposure Reactome pathway analysis of heavy ethanol exposure-sensitive EV-enriched RNA transcripts; paired *t*-test, *p* < 0.05; Hedges’ g > 0.4 with 95% confidence interval that did not cross ‘0’.

## Data Availability

Raw and processed sequenced data files are deposited in NCBI GEO under accession number GSE214545. The ‘R’ code generated by DDC for this study is available on GitHub (https://github.com/daehyukchung/EV_Cell_Transcriptomic.git).

## Abbreviations

FASD: Fetal alcohol spectrum disorders
EtOH: Ethanol
EV: Extracellular vesicle
miRNA: MicroRNA
NSC: Neural stem cell
WGCNA: Weighted gene co-expression network analysis
