# Supplementary figures and images for: Sex differences in the transcriptome of extracellular vesicles secreted by fetal neural stem cells and effects of chronic alcohol exposure
Source: Biol Sex Differ. 2023 Apr 15;14:19. doi: 10.1186/s13293-023-00503-0 (PMC10105449; doi:10.1186/s13293-023-00503-0)

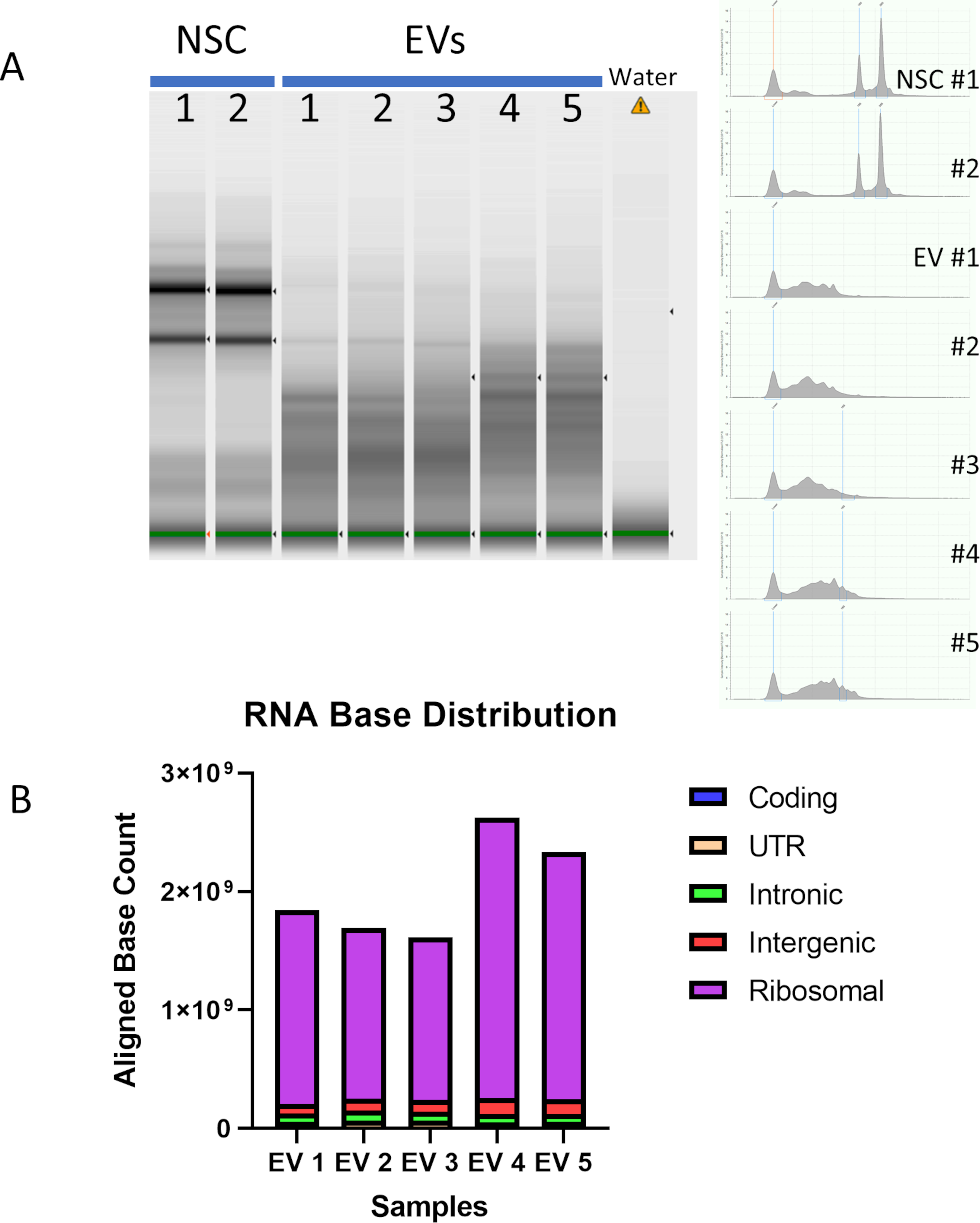

Supplement: Supplementary file 1 — Additional file 1: Fig. S1. RNA quality analysis and Aligned RNA Base Distribution without Ribosomal RNA Depletion. A) Agilent tape station analysis of RNA size distribution in cell-of-origin NSCs and secreted EVs. B) Preliminary RNAseq study of total RNA library prepared without ribosomal RNA depletion of 5 EV samples. In the absence of ribosome RNA depletion, the majority of reads map to ribosomal RNAs. [file 13293_2023_503_MOESM1_ESM.tif]

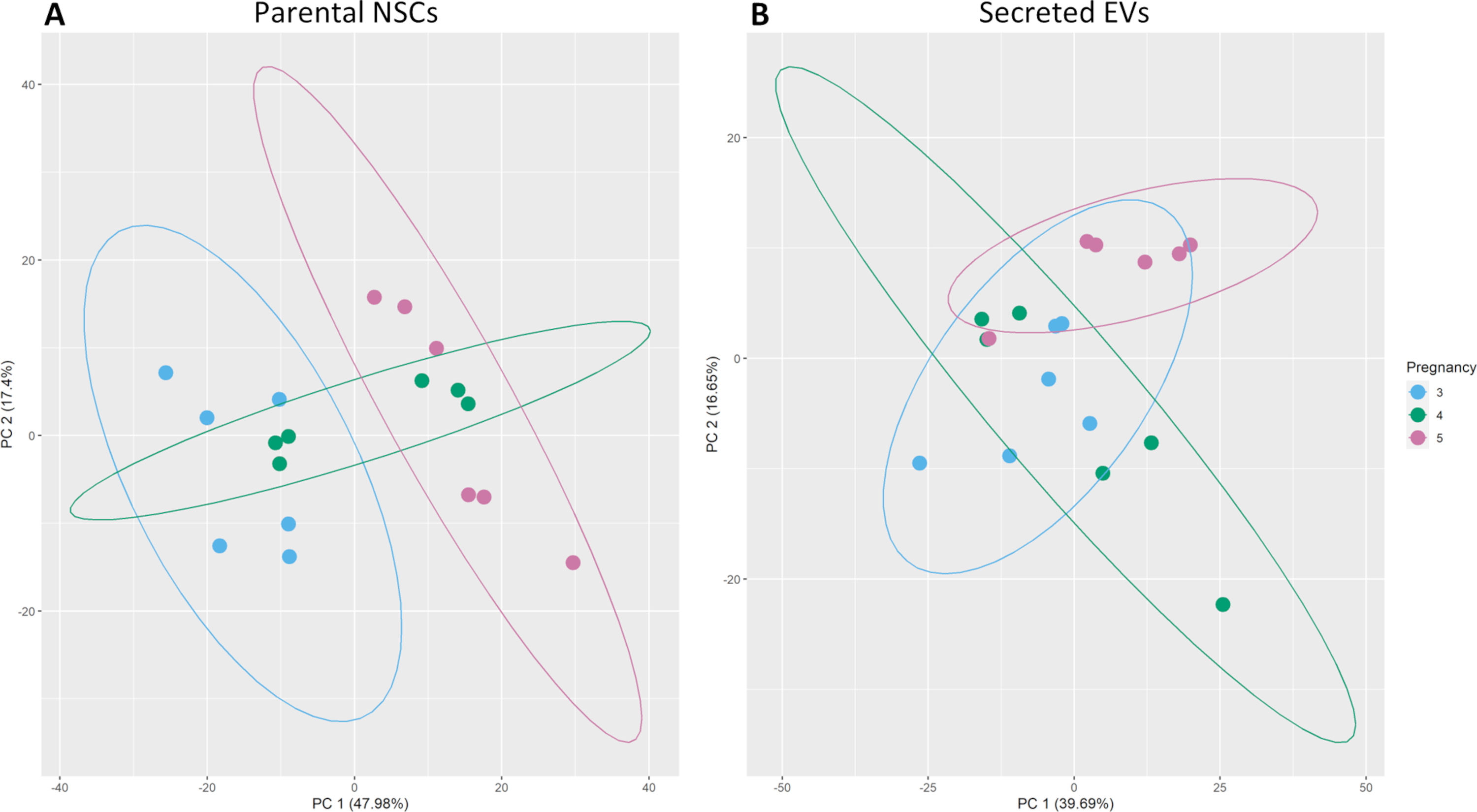

Supplement: Supplementary file 2 — Additional file 2: Fig. S2. Principal Component Analysis of Cell and EV Samples Grouped by Pregnancy. Principal component analysis was performed on the 500 most variant RNA transcripts from 18 NSC parental cell samples (S2A) and their corresponding EV samples (S2B). This analysis shows that parental NSC samples, but not their secreted EVs, could be partly segregated within the 1st two principal components, by their pregnancy identity (i.e., which pregnant dam the fetal cells were derived from). This finding guided this study’s use of a repeated measures experimental design with pregnancy ID as a within-subjects factor for parametric statistical analyses. [file 13293_2023_503_MOESM2_ESM.tif]

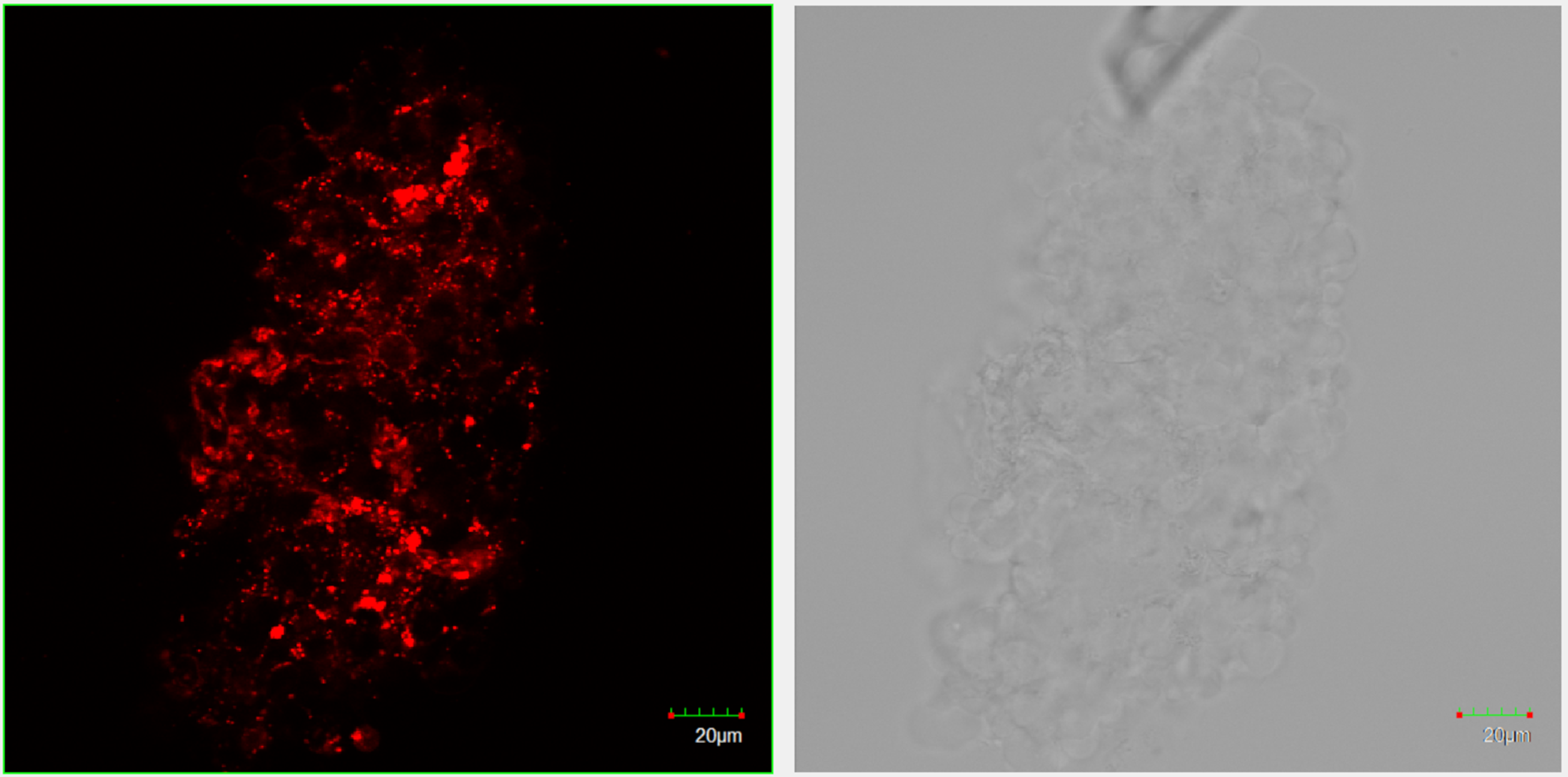

Supplement: Supplementary file 3 — Additional file 3: Fig. S3. Direct Fluorescent Labeling of NSCs with PKH26. Confocal photomicrograph of NSCs that were directly labeled with PKH26 as a positive control with a paired phase-contrast image of a single neurosphere; PKH26-labeled NSCs are shown in red. [file 13293_2023_503_MOESM3_ESM.tif]

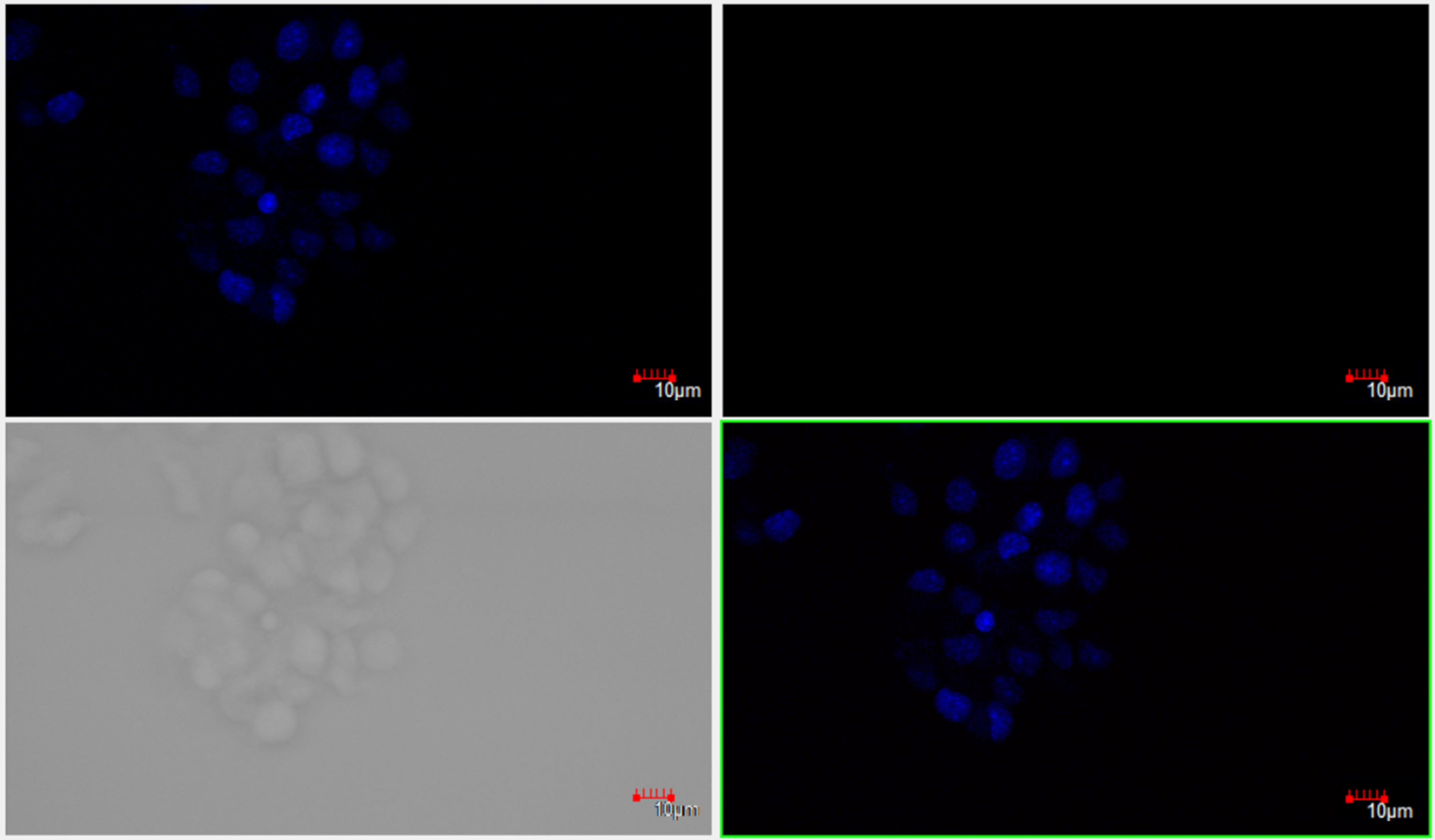

Supplement: Supplementary file 4 — Additional file 4: Fig. S4. Control for Specificity of Fluorescent Labeling Protocol for EVs. Confocal photomicrograph of a negative control, naïve NSCs administered culture medium spiked with PKH26 dye, but subjected to the identical labeling and filtration process as that used for labeling of isolated EVs. This study shows that residual dye is removed by the purification process for EV labeling, and that any fluorescence in recepient cells is due to uptake of labeled EVs (as shown in Fig. 2). NSC nuclei are counter-stained with DAPI (blue fluorescence). [file 13293_2023_503_MOESM4_ESM.tif]

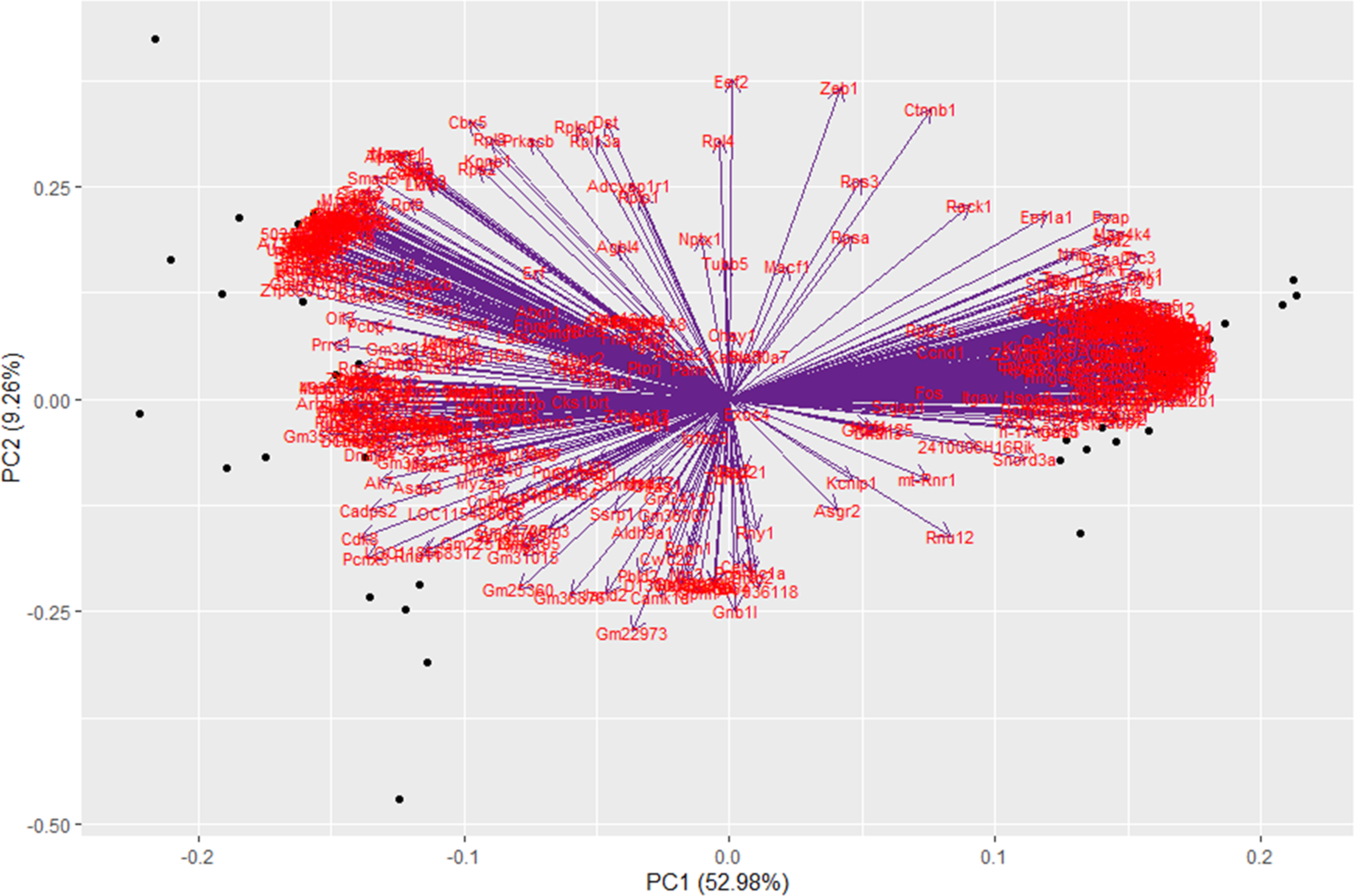

Supplement: Supplementary file 5 — Additional file 5: Fig. S5. RNA transcripts as contributing variables to PCA. Principal component analysis was performed on the 500 most variant RNA transcripts from 18 NSC parental cell samples and their corresponding 18 EV samples. This figure labels RNA transcripts (a full list in Additional file 8: Table S2) that are contributing variables to the principal component analysis, and is supplemental to Fig. 3A, which showed that the samples could be segregated with the 1st principal component, by their sample type (whether the RNA transcripts are from EV or cell sample). [file 13293_2023_503_MOESM5_ESM.tif]

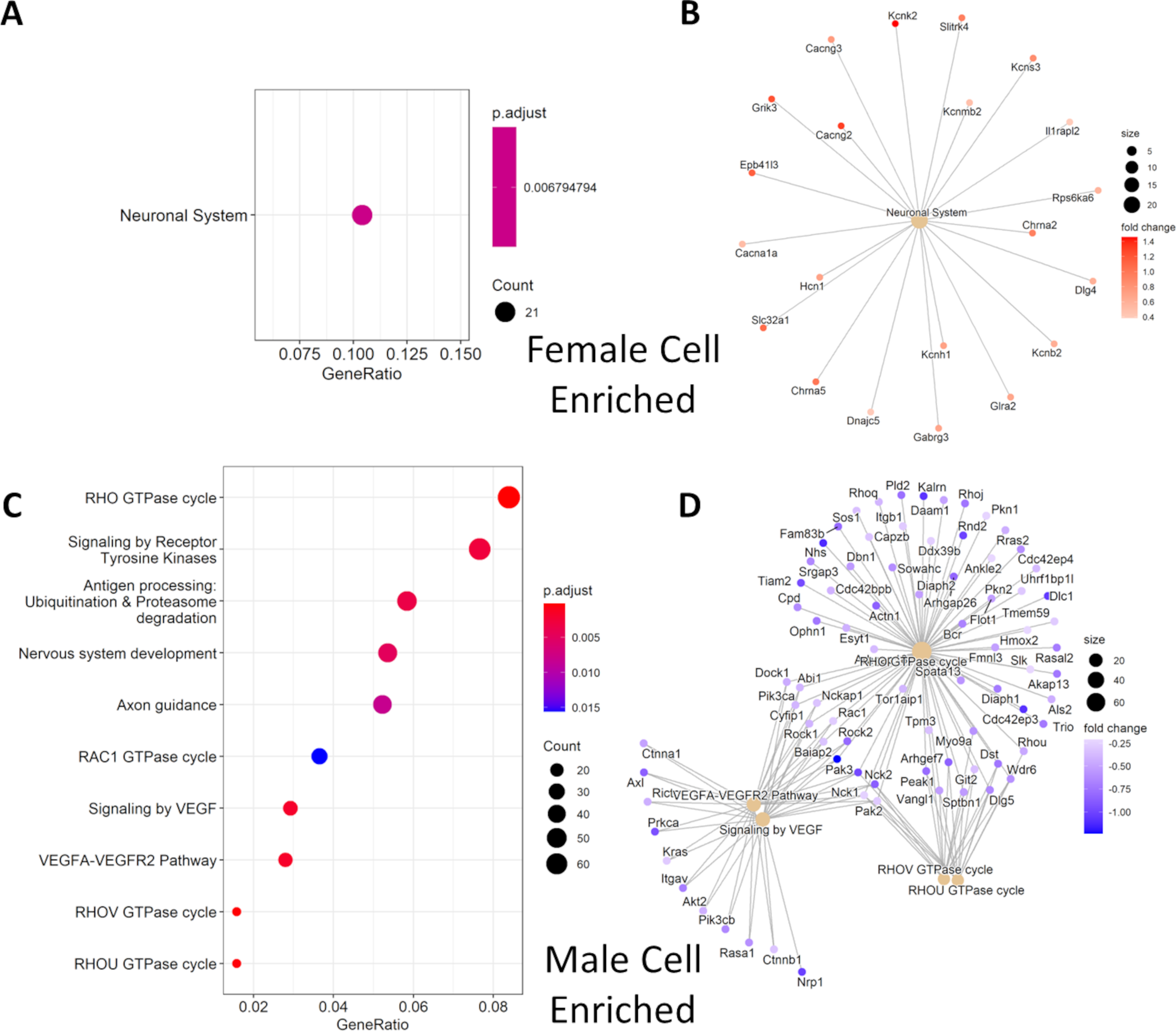

Supplement: Supplementary file 6 — Additional file 6: Fig. S6. Pathway Overrepresentation Analysis of Enriched RNA Transcripts in Cell Samples by Sex. A,C) Dot plot depicting pathways related to significantly altered genes (adjusted p < 0.05) enriched in A) female cell samples relative to male cell samples, C) male cell samples relative to female cell samples, as revealed by ReactomePA. The plot presents overrepresented pathways, ordered by gene ratio, the proportion of differentially expressed genes/transcripts within an ontology term. The size of each dot denotes number of genes/transcripts in a pathway that were contained within this dataset, while the color of each dot encodes the Benjamini and Hochberg-adjusted p-value for significance of pathway overrepresentation. n = 9 female cell samples, 9 male cell samples. B,D) The figures graphically represent the relationship between enriched pathways and their associated genes/transcripts. Significantly altered genes (adjusted p < 0.05) were selected for this analysis. Pathways that reached a Benjamini–Hochberg false discovery rate-adjusted p-value criterion of < 0.05 were selected. The size of each filled central circle represents the number of transcripts in a pathway that were overexpressed in female cells (B), or male cells (D). The color of each dot associated with that pathway denotes the fold change for that transcript in female relative to male. [file 13293_2023_503_MOESM6_ESM.tif]
